# Exploring the landscape of focal amplifications in cancer using AmpliconArchitect

**DOI:** 10.1038/s41467-018-08200-y

**Published:** 2019-01-23

**Authors:** Viraj Deshpande, Jens Luebeck, Nam-Phuong D. Nguyen, Mehrdad Bakhtiari, Kristen M. Turner, Richard Schwab, Hannah Carter, Paul S. Mischel, Vineet Bafna

**Affiliations:** 1 Department of Computer Science and Engineering, University of California at San Diego, 9500 Gilman Drive, La Jolla, CA 92093 USA; 2 Bioinformatics and Systems Biology Program, University of California at San Diego, 9500 Gilman Drive, La Jolla, CA 92093 USA; 3 Ludwig Institute for Cancer Research, University of California at San Diego, 9500 Gilman Drive, La Jolla, CA 92093 USA; 4 Department of Medicine, Division of Hematology-Oncology, School of Medicine, University of California at San Diego, 9500 Gilman Drive, La Jolla, CA 92093 USA; 5 Department of Medicine, Division of Medical Genetics, University of California at San Diego, 9500 Gilman Drive, La Jolla, CA 92093 USA; 6 Moores Cancer Center, University of California at San Diego, 9500 Gilman Drive, La Jolla, CA 92093 USA; 7 Department of Pathology, University of California, San Diego, 9500 Gilman Drive, La Jolla, CA 92093 USA

## Abstract

Focal oncogene amplification and rearrangements drive tumor growth and evolution in multiple cancer types. We present AmpliconArchitect (AA), a tool to reconstruct the fine structure of focally amplified regions using whole genome sequencing (WGS) and validate it extensively on multiple simulated and real datasets, across a wide range of coverage and copy numbers. Analysis of AA-reconstructed amplicons in a pan-cancer dataset reveals many novel properties of copy number amplifications in cancer. These findings support a model in which focal amplifications arise due to the formation and replication of extrachromosomal DNA. Applying AA to 68 viral-mediated cancer samples, we identify a large fraction of amplicons with specific structural signatures suggestive of hybrid, human-viral extrachromosomal DNA. AA reconstruction, integrated with metaphase fluorescence in situ hybridization (FISH) and PacBio sequencing on the cell-line UPCI:SCC090 confirm the extrachromosomal origin and fine structure of a *Forkhead box E1* (*FOXE1*)-containing hybrid amplicon.

## Abstract (web summary)

Focal amplifications are prevalent in many cancer genomes. Here, the authors present AmpliconArchitect (AA), a computational tool for reconstructing their architecture, and reveal an extrachromosomal origin for focal amplifications, including hybrid human-virus elements in HPV-mediated cancers.

## Introduction

Cancer is marked by somatic DNA lesions. While these include small nucleotide changes, and chromosomal aneuploidies, focal amplifications of smaller regions are also a prominent signature in a large proportion of human cancers^1^. Focally amplified regions often involve the juxtaposition of rearranged segments of DNA from distinct chromosomal loci into a single amplified region^2–8^. While common, focal amplifications present a mechanistic challenge—how do multiple regions from one or more chromosomes rearrange together in cancer? Our team recently showed that focal amplification in nearly half of the samples across a variety of cancer types can be explained by circular, extrachromosomal DNA (ecDNA) formation^9^. Furthermore, ecDNA formation can dramatically change the outlook for tumor evolution even as compared with other types of somatic mutations^10^. Due to this renewed understanding, there is an urgent need for tools to study the biological properties of ecDNA, and more importantly facilitate ecDNA-based techniques for cancer treatment and diagnostics. Specifically, tools to elucidate the structure of ecDNA can provide insights into the mechanisms of oncogene amplification and evolution through complex rearrangements.

An “amplicon” is defined as a set of non-overlapping genomic “intervals” connected to each other and amplified, and an “amplicon structure” as an ordered arrangement of genomic “segments” within the amplicon. An amplicon interval may be partitioned into multiple genomic segments depending on the rearrangement breakpoints within the amplicon structures. We recently found that oncogenes amplified on ecDNA are often part of highly rearranged amplicons, which may juxtapose regions from different chromosomes. Traditional structural variant (SV) analyses cannot decipher complex rearrangements^11–14^. The few methods that extend the analysis, chain together breakpoints into paths and cycles, but often do not reconstruct the amplicon in the specific region of interest, and do not provide a comprehensive view of alternative structures^15–22^. Reconstruction remains challenging due to extreme variability in copy counts (5 × −200 ×) and sizes (100 kbp–25 Mbp) of amplicons, samples containing heterogeneous mixture of multiple amplicon structures, and inaccuracy of SV identification.

**Fig. 1:**
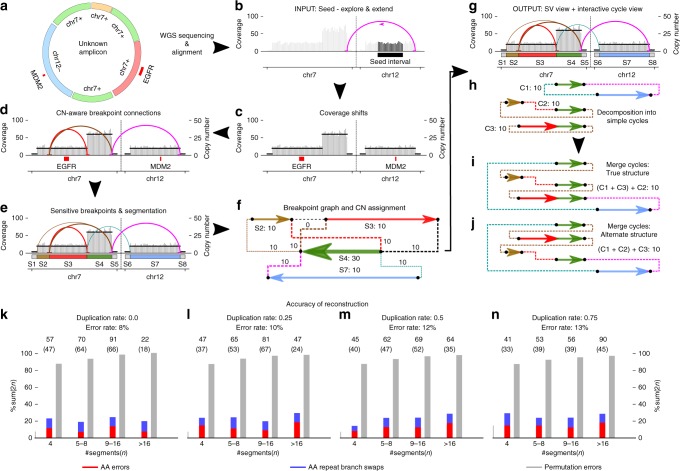
Schematic of AmpliconArchitect (AA). AA takes as input: **a** aligned whole-genome sequencing data from a sample with an amplicon, and **b** a seed interval from the amplicon. It automatically searches for and identifies other intervals that are part of the same amplicon; **c** Next, AA identifies breakpoints by segmenting intervals at positions with a sharp change in copy number, or **d** containing a cluster of discordant paired-end reads. Finally, **e** AA refines breakpoint locations and partitions the intervals into smaller segments. **f** The collection of segments and breakpoints is used to generate a breakpoint graph and a balanced flow approach is used to refine segment copy numbers. Arrows represent the orientation of a segment from lower to higher coordinate. **g**, **h** The entire graph describes a breakpoint signature and a succinct “SV view” of the amplicon, which is also decomposed into short basis cycles in the “Cycle view”. See Supplementary Figure 1 for detailed description. **i**, **j** Alternative merging of the short cycles with overlapping segments can generate multiple amplicon architectures consistent with the short-read data. **k**–**n** Amplicon reconstruction on a variety of simulations showed high fidelity of reconstruction (red bar, 11% error) relative to a random “permutation predictor” (gray bar, 85% error). Swaps (blue bars) represent cases with alternative structures supported by the data. Numbers above each bin represent total number of simulations in the bin and numbers in parenthesis represent number of simulations reconstructed without errors. Source data are provided as a Source Data file

We describe AmpliconArchitect (AA), a tool for reconstruction of ecDNA amplicon structures using whole-genome sequencing (WGS) data (Fig. 1a–j, Methods 1 and 2) that overcomes these difficulties by providing a versatile representation of an amplicon that encodes all supported structures and provides a framework for algorithmic reconstruction of possible structures. Analysis of AA-reconstructed amplicons in a pan-cancer dataset reveals novel properties of copy number amplifications in cancer. In viral-mediated cancer samples, we identify many amplicons with hybrid, human–viral ecDNA. AA reconstruction, integrated with metaphase FISH and PacBio sequencing on the cell-line UPCI:SCC090 confirm the extrachromosomal origin and fine structure of a *FOXE1*-containing hybrid amplicon.

## Results

### Overview of the AA algorithm

AA requires short-read paired-end WGS data aligned to the reference genome. AA automatically infers the read length, insert size distribution, depth of coverage, variability in coverage, and chromosome ploidy relative to the whole-genome and dynamically adjusts its parameters based on these inferences (Methods 2Ai).

**Fig. 2:**
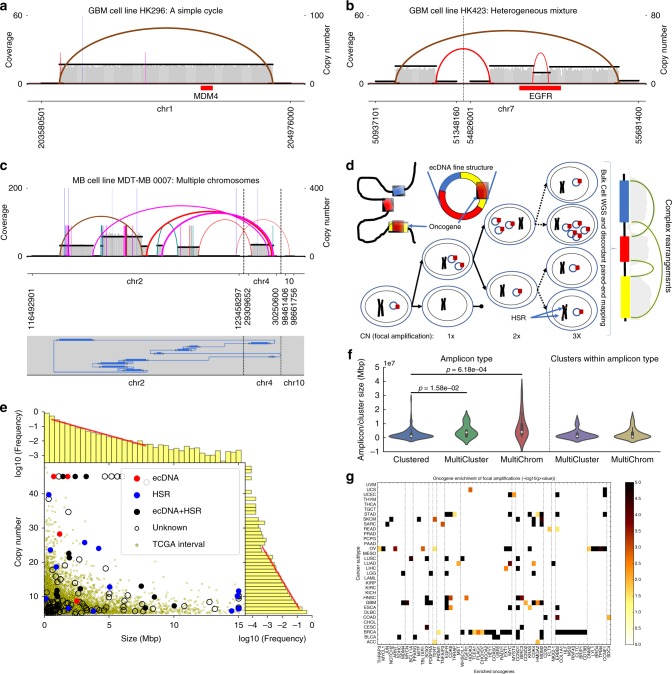
Structural variant (SV) view of AmpliconArchitect (AA) reconstructions. The SV View of reconstructed amplicon structures shows **a** simple cycles; **b** heterogeneity with amplicons containing *Epidermal Growth Factor Receptor* (*EGFR*) VIII deletion, as well as the intact *EGFR*; and **c** SV view and Cycle view of the complex medulloblastoma multi-chromosomal amplification. **d** A model for extrachromosomal DNA (ecDNA)-mediated focal amplification. **e** AA amplified intervals compared against 12,162 amplified TCGA intervals shows significant overlap. The interval sizes (mean 1.74 Mb) and copy numbers (mean 3.16 copies) are exponentially distributed, with no clear distinction between homogenously staining region (HSR) and ecDNA amplicons (Supplementary Data 4). **f** Amplicons containing multiple genomic regions from the same chromosome (MultiCluster) or multiple chromosomes (MultiChrom) are significantly larger in size (*p-*value < 0.016, Wilcoxon Rank-Sum test) than amplicons containing intervals from a single chromosomal region (Clustered). However, the size distribution of individual intervals in clustered amplicons is similar to the size distribution of intervals in MultiChrom or MultiCluster amplicons (Supplementary Data 4). **g** Heatmap of negative log *p*-values (Bonferroni-corrected Poisson Binomial) showing enrichment of 59 oncogenes in amplifications in 33 cancer types. Source data are provided in a Source Data file

Given mapped reads and a seed interval, AA automatically searches for other intervals participating in the amplicon (Fig. 1a, b, Methods 2Bi), and then performs a carefully calibrated combination of copy number variant (CNV) analysis^23^ and SV analysis (Fig. 1c–e, Methods 2Bii). For algorithmic prediction of the amplicon structures, AA uses SV signatures (e.g., discordant paired-end reads and CNV boundaries) to partition all intervals into segments and build a breakpoint graph (Methods 2Biii). It assigns copy numbers to the segments by optimizing a balanced flow on the graph^24^ (Fig. 1f, Methods 2Biv). As short reads may not span long repeats, they cannot disambiguate between multiple alternative structures. AA addresses this in two ways. First, it creates a succinct “SV View” displaying the details of the breakpoint graph, including raw SV signatures, coverage depth, copy number segments, and discordant genomic connections (Fig. 1g, Supplementary Fig. 1a). The SV view by itself is often informative to the user for manual interpretation of the amplicon structure. Second, AA decomposes the graph into simple cycles, and provides a “Cycle View” to intuitively visualize the segments of the cycles in the context of the SV view, showing their genomic locations (Fig. 1h, Supplementary Fig. 1b, Methods 2Bv). The AA Cycle View provides a feature to interactively merge the simple cycles and explore candidate amplicon structures (Figs. 1i, j, 2c, Supplementary Fig. 2-5).

### Datasets

AA was tested on three datasets: (i) a benchmarking dataset with simulations and previously validated samples; (ii) a pan-cancer dataset with low-coverage WGS (sample set 1); and, (iii) cervical cancer sets with samples from the Cancer Genome Atlas project^25^ (sample set 2). The simulations and sample data are described in detail in the following sections. These data included read lengths between 50 bp and 150 bp, variable insert sizes between 150 bp and 700 bp, and coverage ranging from 1 × to 32 ×. We recommend coverage between 5 × − 10× for optimal performance in terms of accuracy and speed. By default, AA downscales datasets with high coverage to 10 × .

### Benchmarking AA on simulations and validated amplicons

A robust amplicon reconstruction tool should predict the amplicon structure for a diverse set of focal amplifications observed in cancer in a high-throughput manner. A complete validation of entire structures would require ultra-long (Mbp) read fragments or isolation of amplicons from the rest of the genome. Moreover, multiple experiments would be needed to test AA effectively on the diversity of reconstructed amplicon structures (e.g., Fig. 2a–c, Supplementary Fig. 2-5, figshare). Therefore, we developed a simulation-based benchmarking strategy and error model to quantify the accuracy of predicted structures.

We simulated a diverse set of 1248 amplicons, including rearrangements with varying levels of copy number (4 × −32 × ), size (40 kbp–10 Mbp), number of rearrangements (0–16), duplication probability (0–0.75), and sequencing coverage (1 × −32 × ) (Methods 4). Here duplication probability refers to the probability of duplication of a segment of the amplicon when simulating the amplicon structure through iterative rearrangements; other events included inversions, translocations and deletions of amplicon segments (Methods 4). To measure the accuracy of the complete reconstruction, we developed a novel metric based on a “graph edit distance”, described informally by the number of operations required to transform the predicted amplicon structure into the true structure (Methods 5, Supplementary Fig. 6). The metric partitions the graph operations into two categories: (i) number of errors caused by AA and (ii) number of swaps across repeat branches indicative of the number of cycle merging operations a user would need to perform to obtain the entire structure. We observed that a mispredicted rearrangement, e.g., a missing edge, will simultaneously affect at least two adjacent segments and increase the graph edit distance by two (Methods 5B). We tested a naive “permutation predictor”, which picks a random order of the amplicon segments from the copy number profile and, indeed, found that the average edit distance of randomly predicted structures to be close to the twice the number of segments in the amplicon structure (Fig. 1k–n). To provide a standard yardstick for assessing our error rate, we normalized the edit distance of the predicted amplicon structures by twice the total number of segments in the amplicon and contrasted against the permutation (Methods 5B). This metric reported that even on our wide-ranging simulations, AA had an error rate < = 11%, averaging about one error per nine rearrangements (Fig. 1k–n, Supplementary Fig. 7). A high fraction of samples was reconstructed perfectly (parenthesized values), although performance decayed slightly when the amplicon was simulated with higher probability of duplication of amplicon segments. AA showed robust performance with changing amplicon size and copy number (Methods 6, Supplementary Fig. 8).

We also compared AA against previous studies which examined complex rearrangements in small sets of cancer samples and validated individual rearrangements using PCR. Specifically, L’Abbate et al.^4^ and Sanborn et al.^20^ experimentally validated a combined 109 breakpoints with number of supporting reads at least 20% of the highest copy, and SV size > 400 bp, and manually presented structures encompassing 103 of them. AA automatically detected 107 of 109 edges and predicted an additional 36 edges as part of a high copy cycle missed by the previous groups (Methods 3, Supplementary Section 1, figshare).

### ecDNA model for focal amplifications in a pan-cancer dataset

We further applied AA to sequencing data of 117 cancer samples (sample set 1) (supplemented by 18 replicates and drug-treated variants) from 13 cancer types, originally described in Turner^9^ (Methods 7, Supplementary Data 1). In querying matched normal samples of the TCGA data, we observed that <1% of the samples would show a germline amplification of size >100 kbp and copy number >5 × (Supplementary Data 2). Therefore, to select seed intervals, we used the CNV tool ReadDepth^26^ (Methods 1, Supplementary Data 3) to identify 255 focally amplified intervals in 55/117 samples with size >100 kbp and copy number >5 × . Using the 255 intervals as seeds, AA reconstructed 135 non-overlapping amplicons, each containing one or more seeds (Supplementary Data 4, figshare). Overall 104 of 255 seeds resulted in single-interval amplicons whereas the remaining 154 seeds were merged into 31 amplicons (Supplementary Fig 9). We observed a range of amplicon structures including simple cycles, heterogeneous mixtures of related structures, a breakage-fusion-bridge amplification, and highly rearranged amplicons with intervals from one up to three chromosomes (Fig. 2a–c, Supplementary Data 4, figshare). The number of detected rearrangements (breakpoint edges) per amplicon ranged from 0 to 49 with average of 4.9 rearrangements per amplicon; this is likely to be an underestimate, due to the low-coverage sequencing data.

Typical mechanisms invoked for copy number (CN) amplification rely on repeated breaks at fragile sites followed by duplication events. We used AA to test an alternative model (Fig. 2d), which (a) starts with breaks at random sites, followed by ecDNA formation; Poisson random breaks result in an exponential distribution of segment lengths. (b) Aggregation of ecDNA create larger, highly rearranged, multi-interval, structures. (c) Replication and independent segregation of ecDNA create cells with a diversity of copy numbers; however, (d) positive selection for higher copy numbers due to proliferative elements (e.g., oncogenes) on ecDNA result in amplification without the need for repeated breakpoint use and duplication events; (e) oncogenes could be expressed in a tissue-specific manner providing selective advantage to different tumor subtypes. Therefore, amplicons sampled from specific tumor subtypes could be enriched in specific oncogenes, while being structurally quite different; finally, (f) ecDNA structures reintegrate into non-native chromosomal locations as homogenously staining regions (HSRs), while maintaining their structure across cell passages.

The model suggests that many chromosomally located amplicons may also have originated as ecDNA. As sequence data are not sufficient to directly test this hypothesis, we analyzed AA predictions in 45 out of 135 amplicons where metaphase FISH experiments had been performed by Turner et al.^9^. We used the simple criteria that presence of a cycle of length ≥10 kbp and copy count ≥4 in the AA reconstruction was evidence of an ecDNA origin. Low coverage (< 1 ×) in our WGS data implied that we could get false negatives due to missed discordant edges, especially when the FISH ecDNA counts were low. To quantify this, we selected a threshold *t*, and called the sample FISH positive if the average number of ecDNA per cell was ≥*t*. For *t* = 1 (at least one ecDNA per cell on average), 11 of the 17 samples with cyclic AA reconstructions were FISH positive, whereas only 6 of 28 non-cyclic reconstructions were FISH positive (*p*:0.005, Fisher's exact test). The results remained significant for a range of values for *t* (0.5 ≤ *t* ≤ 2). (Supplementary Data 5). The AA prediction of amplicons with ecDNA origin will further improve with higher coverage (5 × −10 ×) WGS data and larger numbers of FISH samples, and lead to better criterion for calling ecDNA from sequence.

To test the other tenets of this model, and get statistically more meaningful numbers, we expanded the 135 AA amplicons in sample set 1 with 12,162 somatically amplified intervals in 2513 TCGA^25^ samples determined by CNV arrays (Methods 8). Importantly, intervals from the AA amplicons showed a significant overlap with the TCGA intervals (Methods 9, *p*-val :1.1e–8).

Consistent with tenet (a), the TCGA interval size and copy number both followed exponential distributions with mean 1.74 Mbp and 3.16 copies, respectively (Methods 10; Fig. 2e, Supplementary Fig. 10). Of the 135 amplicons reconstructed by AA, 104 amplicons consisted of a single interval from the genome, whereas 31 amplicons contained multiple intervals. The single-interval amplicons included nine amplicons that had a simple structure with a single discordant edge connecting the end of a segment to the start of the segment. This sequence signature can be a result of either tandem duplications with exact breakpoints or a circular ecDNA formed by circularization of the segment. Many of the single-interval amplicons had complex structures with up to 12 rearrangements and segments with multiple copy number states (see figshare). The 31 multi-interval amplicons included 17 “MultiChrom” amplicons containing intervals from multiple chromosomes, whereas the other 14 amplicons were “MultiCluster” amplicons containing multiple intervals from a single chromosome. Individual intervals within multi-interval AA amplicons were similar in size to single-interval amplicons (Fig. 2f). However, in support of tenet (b), we observed that MultiCluster amplicons with mean size 4.7 Mbp and MultiChrom amplicons with mean size 7.7 Mbp were, on average, larger in size than the single-interval amplicons, which had a mean size of 2.4 Mbp (*p*-values = 1.58e–2, 6.18e–4, Fig. 2f, Methods 11, figshare). In support of tenets (c), (d), we had previously shown an increase in the copy number heterogeneity as well as an enrichment of oncogenes in ecDNA^9^. Tenet (e) of the model postulates that ecDNA drive tumor growth through the amplifications of oncogenes that confer a selective advantage to a tumor subtype. Indeed, we found that amplifications of 59 distinct oncogenes were specifically enriched in 19 of 33 cancer types in the TCGA sample set (Fig. 2g, Methods 12). For example, *MDM4* and *EGFR* were enriched in glioblastoma, *MYC* and *ERBB2* were enriched in breast cancer whereas *MDM2* was enriched in both. In turn, a significant portion of the enriched oncogenes manifested in the amplicons of the corresponding cancer types from sample set 1. Limiting the analysis to the 48 enriched oncogenes in 10 TCGA subtypes that were present in sample set 1, we found that amplicons in four cancer types contained 18/48 oncogenes enriched in the corresponding TCGA cancer types, whereas in four more cancer types, the corresponding TCGA samples did not show any enriched oncogenes (Supplementary Data 6).

**Fig. 3:**
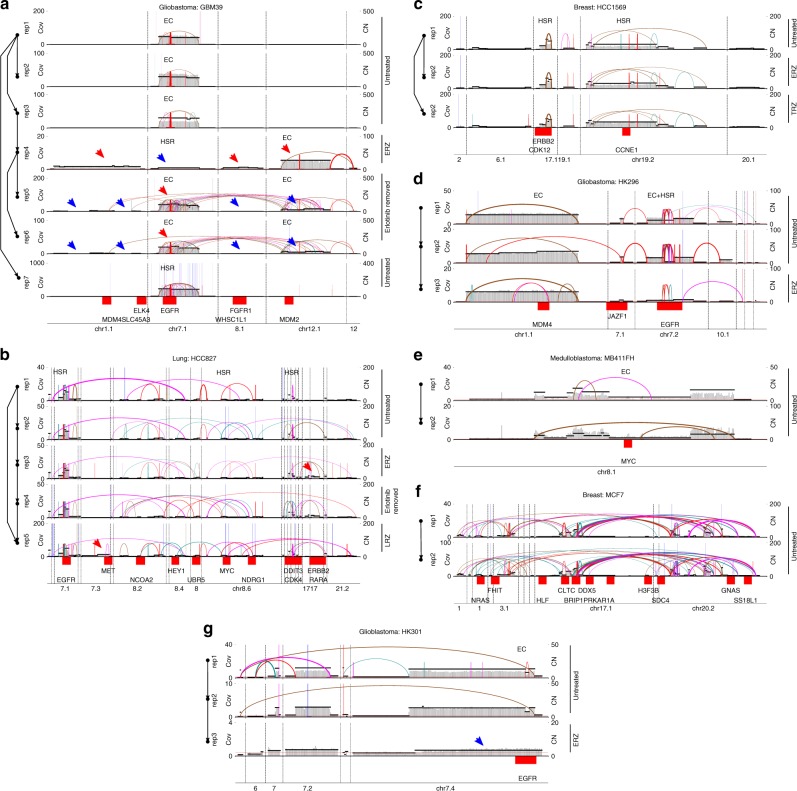
AmpliconArchitect (AA) amplicons evolving over time or in response to drug treatment: **a** GBM39: patient-derived xenograft (PDX), glioblastoma; **b** HCC827: Cell-line, lung; **c** HCC1569: Cell-line, breast; **d** HK296: Cell-line, glioblastoma; **e** MB411FH: Cell-line, medulloblastoma; **f** MCF7: Cell-line, breast; **g** HK301: Cell-line, GBM. For each sample, one row per replicate shows a combined structural variant (SV) view of all amplicons including intervals amplified in other biological replicates of that sample. The left axis shows replicate ID and passage of cell line or PDX, whereas right axis shows the condition of the replicate: Untreated, undergoing drug treatment or after drug removal; ERZ: erlotinib resistant; LRZ: lapatinib resistant; TRZ: trastuzumab resistant. Within each replicate, known locations of oncogenes, EC (ecDNA), HSR or EC + HSR, as determined by FISH, are indicated in the corresponding replicate row. Red and blue arrows, respectively, indicate a gain and loss in copy number or formation of new amplicons with respect to parent cell line or PDX

In support of tenet (f), we start by noting that both intra-chromosomal (HSR) and extrachromosomal (ecDNA) amplicons occurred with a large variation of size and copy number (Fig. 2e), albeit with HSR elements being somewhat larger and lower in copy number. Importantly, detailed AA reconstructions showed that amplicons preserved their structures within biological replicates but evolved over time, in response to drug treatment, and in transition from ecDNA to HSR and back^9^ (Fig. 3). Amplicons could change their location spontaneously from ecDNA to HSR or in response to drug (Fig. 3a). Sometimes, new amplifications or structural changes appeared with changing drug environment, e.g., *MDM2*, *MDM4*, *FGFR1* and *WHSC1L1* in GBM39 and *ERBB2* and *MET* in HCC827 (Fig. 3a, b).

To test whether neighboring chromosomal features or functional elements outside the oncogenes played a role in amplicon formation or tumor growth in multiple samples, we measured the size of the overlap between amplified intervals containing the top three oncogenes—*EGFR*, *MYC*, and *ERBB2*. For each oncogene, and each pair of samples focally amplifying the oncogene, we measured the size of the overlap to evaluate the hypothesis that the size of the overlap was significantly larger than in a null model in which overlaps are obtained from a random choice of breakpoints around the oncogene. The QQ plots of pairwise similarity scores indicated that the null hypothesis could not be rejected (Supplementary Fig. 11, Methods 13). Thus, we found no evidence to suggest that recurrent breakpoint use is important for amplicon formation. These results complement previous results^2^, which also reported a lack of association with fragile sites, segmental duplications (SDs) or repetitive elements in regions of complex genomic rearrangements, and they strengthen the case for an ecDNA-based model of CN amplification.

### Chimeric human–viral amplifications in cervical cancer

**Fig. 4:**
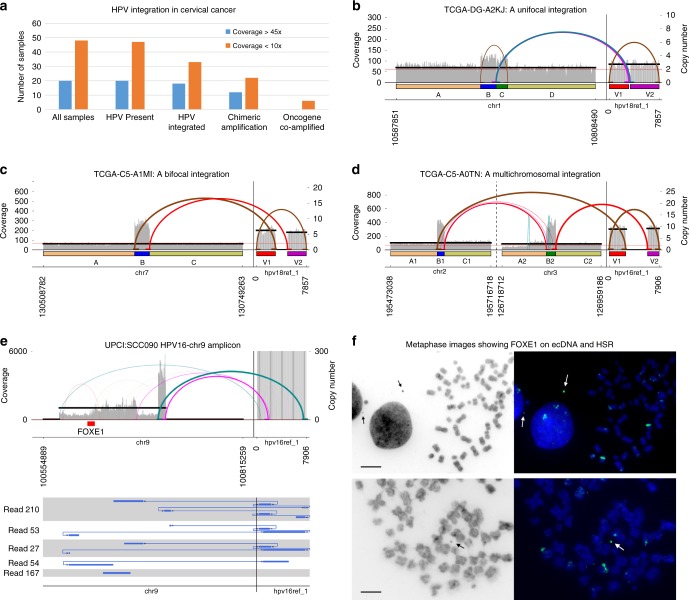
Amplicon structures near viral insertions. **a** Human papillomavirus (HPV) sequence was identified in 67 of 68 tumor whole-genome sequencing (WGS) samples (with genomic integration in 51) compared with 0 of 68 in matched normal blood samples. Forty-one fusion amplicons were reconstructed in 33 samples (Supplementary Data 7, 8). **b** Although 14 of the viral insertions gave a unifocal amplification signature, consistent with viral insertion at a specific genomic location, **c** 32 amplicons showed a bifocal signature. **d** A two-way bifocal signature in sample TCGA-C5-A0TN with two segments from chr2 and chr3 connected to a viral segment in a circular or tandemly duplicated structure with 10 copies. The prevalence of bifocal signatures is suggestive of hybrid extrachromosomal DNA (ecDNA) elements containing virus and human sequence (Supplementary Data 8). **e** AA reconstruction of a complex hybrid structure (>100 kbp) containing the oncogene *FOXE1*. The Cycle view shows paths traced by PacBio reads validating the AmpliconArchitect (AA) structures. **f** Metaphase images of cells in metaphase of UPCI:SCC090 stained with DAPI (left: black, right: blue) and FISH on a *FOXE1* probe (green) indicating the presence of ecDNA (arrow), as well as numerous HSRs containing *FOXE1*. Sizes of the black bars in the DAPI images correspond to 200 μm in the respective DAPI and FISH images

In a second application of AA, we looked at focal amplifications near genomic viral integrations from 68 cervical cancer tumor samples^25^ (sample set 2) with matched normal blood samples (Methods 14, Supplementary Data 7). AA detected human papillomavirus (HPV) genomic sequence in 67/68 tumor samples and none in matched normal. We found HPV integrated into the human genome in 18/20 high-coverage (>30 ×) and 33/48 low-coverage (<10 ×) samples^27^. AA reported that viral integrations induced formation of 41 human–viral fusion amplicons containing both viral DNA and segments from the human genome in 49% (33) of all samples (Fig. 4a, figshare, Supplementary Data 8). Although six fusion amplicons contained an oncogene (three with *MYC*, one each with *ERBB2*, *BIRC3*, and *RAD51L1*), the majority of fusion amplicons contained human sequence from intergenic regions.

The simplest mechanism of viral integration, which we denote as a unifocal integration, consists of the virus inserting itself into the human genome by causing exactly one double-stranded break (Fig. 4b). AA reconstruction revealed a novel bifocal signature where the endpoints of the amplified human interval were flanked by the virus on each side (Fig. 4c). For example, if four-ordered segments ABCD represent a section of the normal human genome and V represents a viral segment, then a fusion amplicon induced by a unifocal integration might result in a structure of the form A[BVC]^n^D. In contrast, we see structures of the form AB[VB]^n^C, reminiscent of a bifocal insertion. A simpler explanation for bifocal signature is a circular extrachromosomal amplification of the form (BV) where V is connected back to B. Only 14 (34%) fusion amplicons displayed a unifocal signature. In contrast, 19 (46%) amplicons displayed a bifocal signature. An additional 12 (29%) amplicons showed a “weak” bifocal signature where only the highest copy segment was flanked by the virus but the virus did not flank neighboring amplified segments with smaller copy numbers (Supplementary Fig. 12, Methods 15, Supplementary Data 8). Thirteen amplicons contained multiple human–viral connections. Sample TCGA-C5-A0TN contained an unusual amplicon with a two-way bifocal signature where two segments from chr2 and chr3 were connected together and the virus in turn flanked the outer end of each segment in a circular or tandemly duplicated structure with 10 copies (Fig. 4d). Through simulations, we concluded that a unifocal integration followed by random rearrangements is unlikely to result in the formation of an amplicon with a bifocal signature (Methods 16, Supplementary Fig. 13). The 62 breakpoints supported by split-reads showed NHEJ or MMBIR signatures similar to breakpoints in sample set 1 (Supplementary Fig. 14).

Akagi et al.^28^ have proposed a looping model where origins of replication on the human genome drive amplification. However, the prevalence of bifocal signatures and multiple chromosomes as part of an amplicon and the ubiquitous presence of the HPV origin of replication alongside the viral oncogenes E5 and E6 in reconstructed amplicons suggest an alternative possibility that the chimeric amplification could be mediated through ecDNA formation. Although episomal virus in its native form has been reported extensively in cancer cells, the AA reconstructions make a compelling case to investigate the presence of fusion human–viral segments in the form of ecDNA. To test this hypothesis, we identified a head and neck cancer cell-line UPCI:SCC090, where the AA reconstruction showed tremendous heterogeneity of structure, but also the prevalence of cycle containing the oncogene *FOXE1*. The AA reconstruction was further confirmed using PacBio WGS where long, single-molecule reads supported multiple breakpoints (Fig. 4e). We observed extensive heterogeneity. For example, PacBio read collections (210, 53, 27), and (54, 27,167) indicate two different cyclic structures supporting AA breakpoints in Fig. 4e, consistent with multiple intra-chromosomal insertions of the amplicon. Metaphase FISH using a probe for *FOXE1* showed its occurrence as ecDNA, as well as multiple non-native chromosomal insertions (Fig. 4f). The existence of multiple *FOXE1*-containing ecDNA and HSR structures is consistent with a common, extrachromosomal origin for these amplicons.

## Discussion

AA is a robust and viable tool for reconstructing possible ecDNA and other focal amplicon structures from short-read data and allows for an interactive exploration of alternative structures. The size of ecDNA (up to 25 Mb) implies that existing single-molecule assembly is also not sufficient for ecDNA reconstruction. Therefore, AA reconstructions on inexpensive short-read data can be used as a template for guiding assembly of longer, single-molecule reads. AA provides a cycle decomposition that explains at least 80% of the genomic content of the amplicon. There can be multiple sources affecting complexity of structure, e.g., recombination of ecDNA elements, rate of tumor evolution, functional elements providing selective advantage. By choosing the cutoff as percentage of genomic content, AA makes the conscious decision to specifically focus on the structures with high abundance as these structures are likely to drive proliferation. Finally, analysis of AA reconstructions on multiple pan-cancer datasets suggests that ecDNA could play an important role in creating complex rearrangements and focal amplifications observed across the spectrum of cancer subtypes.

## Methods

### 1) Seed interval selection: interval merging and copy number threshold

AA requires a seed interval in addition to mapped genomic reads. The seed interval serves as a starting point for AA to search for all connected genomic intervals contained in the amplicon. Here, we pick seed intervals in two different sample sets: (a) WGS of tumor samples across multiple cancer types, (b) WGS of cervical cancer samples infected with HPV.

A) To identify the set of seed intervals in the WGS samples from the pan-cancer dataset, we defined parameters CN_THRESHOLD and SIZE_THRESHOLD for minimum bounds on copy number and size of the interval. Aiming to find a criterion for identifying somatically amplified intervals, we compared the CNV calls for matched tumor and normal samples downloaded from TCGA consisting of 22,376 masked CNV call files from TCGA generated from Affymetrix 6.0 data for 10,494 matched cases. For a given CN_THRESHOLD, define an amplified segment as a single CNV call with copy number greater than CN_THRESHOLD. A sample might have multiple amplified segments adjacent to each other. The size of the amplified segment was simply the number of basepairs in the segment. For each sample, we merged consecutive amplified segments within 300 kbp of each other to create the set of amplified intervals. The size of amplified interval was the sum of sizes of the amplified segments in the interval and the copy number of the amplified interval was the weighted average of the amplified segments weighted by their sizes. We counted the number of amplified intervals in all normal and tumor samples in the TCGA set for values of CN_THRESHOLD in {3, 4, 5, 7, 10} and SIZE_THRESHOLD in {10 kbp, 50 kbp, 100 kbp, 200 kbp, and 500 kbp}. Based on these, we chose the values CN_THRESHOLD = 5 and SIZE_THRESHOLD = 100 kbp for selecting the seed intervals, which resulted in 145 intervals in normal samples and 12,162 intervals in tumor samples, which did not have a corresponding amplification in the normal samples.

Next, we analyzed the WGS reads from the WGS samples from the pan-cancer dataset. We mapped the reads to the hg19 genome from UCSC genome browser^29,30^ and obtained CNV calls using the CNV calling tool ReadDepth CNV software version 0.9.8.4^31^ with parameter false discovery rate = 0.05 and overdispersion parameter = 1. We also obtained the CNV calls for eight normal control samples and created the set of amplified intervals from the CNV call sets for all samples with CN_THRESHOLD = 5 and SIZE_THRESHOLD = 100 kbp. First, we looked at the amplified intervals from the normal samples and found genomic regions, which were amplified in two or more normal samples. These regions were marked as blacklisted regions. As ReadDepth did not report CNV calls for chrX and chrY, we used a previously computed list of recurrent CNVs on the X and Y chromosome reported by Layer et al.^14^.

From the amplified intervals from WGS of tumor samples, we filtered out false intervals using three criteria:

(i) We identified intervals overlapping blacklisted regions and trimmed them to exclude the portions within 1 Mbp of the blacklist regions.
(ii) For each interval, we calculated its average repetitiveness by defining Duke35 repetitiveness score based on the mappability score track from UCSC genome browser^30^. This mappability score track reports the repeat count of each 35-bp window in the reference genome up to copy number 5. We computed the Duke35 repetitiveness score of an interval as the average score of all 35-bp windows in the interval and filtered out intervals with score > 2.5.
(iii) We looked at intervals overlapping regions of SD reported by the human paralog project. For these intervals, we defined an SD-adjusted copy number as the interval copy number downscaled by its average repeat count. In the absence of information regarding actual repeat counts of the SDs, we assumed a repeat count of 2. As a result, the SD-adjusted CN = Interval CN/(1 + Total length of overlapping SDs/length of interval). We only retained amplified intervals if their SD-adjusted CN was >5.

Finally, to correct for the copy number gain due caused due to aneuploidy, we required that the difference in copy number of the interval and the median copy number of the chromosomal arm should be at least 3. Specifically, for amplicons in chromosomes with reference copy number of two, the copy number cutoff was 2 + 3 = 5 = CN_THRESHOLD. We applied these filters on the ReadDepth calls for the 117 WGS tumor samples to obtain 255 intervals in 55 samples.

B) For detecting chimeric human–viral amplification in cervical cancer samples, we created a combined reference genome consisting of the human chromosomes and the viral reference genome and aligned reads to this combined reference (Methods 13). We selected the viral genome as the seed interval. This was a highly selective criterion for selection of the seed interval, which allowed us to perform a more sensitive search for amplicons. As a result, there was no initial cutoff on copy number or size of the seed interval. The cutoffs were chosen at the end of final reconstruction.

### 2) AA methods

The AA pipeline starts with the seed interval and mapped reads and performs multiple steps including search for amplicon intervals, detection of genomic rearrangements, construction of breakpoint graph, decomposition of the graph into simple cycles, and visualization of the cycles. To perform these steps efficiently and accurately, AA implements and uses multiple low-level modules. Here, we briefly describe the implementation of AA pipeline and the low-level modules. The AA software described here may be downloaded from https://github.com/virajbdeshpande/AmpliconArchitect.

A) Low-level modules:

(i) Sequencing parameter estimation: this module estimated the parameters of sequencing coverage and variability as a function of window size, as well as the read and fragment insert lengths of the sequencing library. Given a bam file of mapped reads and a window size ws, AA obtained an initial estimate of the median coverage for 1000 randomly chosen windows from non-blacklisted regions, excluded all windows with coverage = 0 or > 5 times the initial median and recalculated the mean (*μ*_ws_), median (*θ*_ws_), and standard deviation (*σ*_ws_) of window coverages for the given window size. AA computed the coverage for window sizes ws = 10 kbp and ws = 300 bp. Further, it obtained the read pairs from the windows used for estimating the coverage with window size 10 kbp, estimated the fraction *P* of “properly mapping” read pairs and estimated the mean read length *R* and the mean (*µ*(*I*)) and standard deviation (*σ*(*I*)) of the fragment insert length of the properly mapping read pairs as reported by the read aligner in the alignment flags of the Sequence Alignment/Map (SAM) file format.
(ii) CNV boundary detection: this module identified positions of copy number changes in an interval using only the coverage histogram. It first identified an initial list of boundaries of CNV segments based on a histogram with window size 10 kbp and then refined these boundaries through a local search based on a histogram with window size 300 bp within the neighborhood of the initial list of boundaries. To estimate the CNV boundaries for a given interval size, AA used a meanshift procedure adapted from Abyzov et al.^23^. In the meanshift procedure, AA identified the CNV boundaries as the locations of the minima of the Gaussian kernel density estimator indicative of a large change in coverage. Specifically, for each window *i*, define a Gaussian kernel density function *F*_*i*_:
  $$F_i = {\rm norm}\mathop {\sum }\limits_{j \ne i} {\mathrm e}^{ - \frac{{(j - i)^2}}{{2H_{\mathrm b}^2}}} \cdot {\mathrm e}^{ - \frac{{(r_i - r_j)^2}}{{2H_{\mathrm r}^2}}}$$
  Here *j* iterates over 50 neighboring windows on either side of window *i*, *r*_*i*_, *r*_*j*_ are the coverage depths for bins *i* and *j*, *H*_b_ is the bandwidth for bin index and *H*_r_ is the bandwidth for the coverage depth. For bin *i* with size ws and coverage *c*_*i*_, AA set $H_{\mathrm r} = {\mathrm{max}}(2,\,\sqrt {c_i/\theta _{\rm ws}} )\sigma _{\rm ws}$. *H*_b_ iteratively took values in the order (2, 5, 10, 50, 100) as described below. norm represents the constant normalization coefficient. Thus, (∇*F*)_*i*_, the component of the gradient of *F*_*i*_ along the genomic coordinates is:
  $$\left( {\nabla F} \right)_i = \left( {\frac{{\rm norm}}{{H_{\mathrm b}^2}}} \right)\mathop {\sum }\limits_{j \ne i} \left( {j - i} \right) \cdot {\mathrm e}^{ - \frac{{(j - i)^2}}{{2H_{\mathrm b}^2}}} \cdot {\mathrm e}^{ - \frac{{(r_i - r_j)^2}}{{2H_{\mathrm r}^2}}}$$
  AA selected the boundaries between pairs of consecutive windows where (∇*F*)_*i*_ changes from negative to positive, merged all windows between these boundaries into segments and calculated the average coverage *C*_s_ for each segment *s*. AA selected the boundaries where the difference in coverage |*C*_s1_−*C*_s2_| for consecutive segments s1 and s2, was found to be significant as described below. If either segment was smaller than 15 windows, then it required |*C*_s1_−*C*_s2_| > 3*σ*_ws_∙ max(*C*_s1_, *C*_s2_)/*θ*_ws_. AA detected the meanshift boundaries at various scales by iteratively increasing the size bandwidth from 2 to 100 windows while freezing segment boundaries it found significant in each stage. AA obtained an initial high confidence set of CNV boundaries in an interval by searching for the meanshift boundaries in the entire interval with window size ws = 10 kbp. It then refined these boundaries by running the meanshift algorithm with window size ws = 300 bp and from the new call set, picking the new boundary with desired directionality change in coverage and largest difference in coverage of adjacent segments.
  The CNV boundary detection module calculates the average coverage for genomic segments and defines the coverage ratio as ${\mathrm{CR}}_{s} = 2{\mathrm{C}}_{s}/\mu _{ws}.$ The module could be run in two modes. In the sensitive mode, the difference in coverage of adjacent segments |*C*_s1_−*C*_s2_| was considered significant as determined by independent *t-*test of the distribution of window coverage of s1 and s2. In the default mode, the module further filtered out boundaries if the difference in coverage ratios $\left| {\mathrm{CR}}_{s1}-{\mathrm{CR}}_{s2} \right| < {\mathrm{max}} (1,\sqrt {{\mathrm{CR}}_{s1}} ,\,\sqrt {{\mathrm{CR}}_{s2}} )$. The sensitive mode was specifically used for chimeric human–viral amplicons where the virus could have very high copy number as compared with the human intervals due to independent amplification. The default mode was used for all other amplicons in order to focus on amplicon structures that comparable in abundance with the highest copy structure and ignore noise from low copy rearrangements.
(iii) Breakpoint detection: This module took as input one or more intervals, and identified all breakpoints associated with these intervals using discordantly mapping read pairs as follows:
  - Identify all discordant reads mapping to the intervals such that the mate maps either maps to a different chromosome, has unexpected mapping orientation, or maps at location such that the distance between the outermost mapping positions of the read pairs is outside of the range (*µ*(*I*)−3*σ*(*I*), *µ*(*I*) + 3*σ*(*I*)). Cluster reads that map within (*µ*(*I*) + 3*σ*(*I*)) basepairs of each other and have the same mapping orientation.
  - For reads with each cluster identify mapping positions of the mates and create one or more cluster pairs, “biclusters” from read pairs (including secondary alignments) where the first cluster consists of subset of reads from cluster from step (a) and that the second cluster corresponds to mates of these reads that map within (*µ*(*I*) + 3*σ*(*I*)) of each other.
  - For each bicluster, filter reads in repetitive regions with Mapping Quality score (MAPQ) ≤ 5 or satisfying one of the three criteria for filtering out repetitive intervals described in the seed interval selection process (Methods 1).
  - Remove biclusters whose size is smaller than a significance threshold *S* as described below. The size of a bicluster is the number of unique read pairs in the bicluster.
  - Report pairs of breakpoint inferred from bicluster.

The significance threshold for number of read pairs in the bicluster could be chosen from four different options used for specific scenarios: (a) a fixed input parameter (e.g., two read pairs for a sensitive search), (b) minimum number of read pairs determined by the average sequencing coverage, read length, and fragment insert length, (c) minimum number of read pairs for a region with copy number estimated by the coverage ratio of the meanshift segments, or (d) minimum number of read pairs determined by the difference in the coverage ratio across the CNV boundary. The minimum number of read pairs *S* associated with a given CR or difference in CR of segments was calculated as $S_{\rm CR} = P \times {\rm CR} \times \mu _{300} \times (I-R)/2R/D$. Here *D* = 20 was a downscaling factor, which we chose based on observations from our simulations, which suggested that the expected number of read pairs scaled down 20 times provided a classifier with high sensitivity without affecting specificity at multiple copy number states. Selection and hard-coding of the parameter *D* was the only fingerprint of “training” AA based on the simulation “evaluation set”, otherwise all development of AA was done prior to evaluation on the simulated examples.

B) AA pipeline:

AA implemented a series of steps to start from a seed interval and ultimately reconstruct the full structure of the amplicon and provided informative results from each stage:

(i) Interval search: In this step, AA started with the seed interval and iteratively identified the list of intervals belonging to the amplicon. It started by creating a max-heap data-structure storing the seed interval.
  - AA repeated the following steps until the max-heap was empty or after 10 iterations (Fig. 1b).
    In each iteration, AA selected an interval and determined the discordant read pair biclusters in a CN-sensitive fashion and selected biclusters with the mate mapping outside previously seen intervals.
    It then attempted to extend the bicluster by querying whether the extended portion is amplified. A query segment was classified as amplified if it had at least 20% of windows(ws = 10 kbp) with coverage > *θ*_10,000_+ 3*σ*_10,000_, or if it was smaller than 20 kbp and contained at least two discordant edges (bicluster size corresponding to CN = 2). AA then efficiently extended the query bicluster by iteratively doubling the size of the extended portion until the extension is found to be amplified and then iteratively reduced the extension query size by half. If AA was able to successfully extend the query bicluster, then it extended it further by 100 kbp and recorded the extended interval for future iterations.
    After AA recorded all amplified neighbors, AA marked the interval as *seen*, updated the max-heap ordered by the number of discordant read pairs connected to previously seen intervals and greedily picked the interval at the top of the heap for the next iteration.
  - AA reported all amplified intervals from the extension step.
(ii) Interval rearrangements, partition, and visualization:
  - AA calculated all the coverage meanshift boundaries and initial copy number estimates for corresponding segments (Fig. 1c).
  - It then created the list of discordant read pair biclusters with bicluster size thresholds determined by the CN estimates (or differences) (Fig. 1d).
  - Additionally, for meanshift boundaries where it did not find a matching discordant read bicluster, it performed a sensitive local search for discordant read pairs with a bicluster size threshold of just two read pairs (Fig. 1e).
  - Finally, it created the set of genomic locations of all rearrangements with a discordant read pair bicluster or a meanshift boundaries with no matching discordant reads. Using this set of locations, it partitioned all the intervals into sequence edges.
  - For the output from the second stage, AA created a single plot called the SV view, which displayed the interval set with the coverage histogram, initial copy number estimates of the meanshift segments and the discordant read biclusters.
(iii) Breakpoint graph construction: AA used the sequence edge partitions to construct a breakpoint graph^32^ (Fig. 1f).
  - For each sequence edge, it created two breakpoint vertices marking the start and end of the genomic segment and added a sequence edge connecting the two vertices.
  - AA augmented the vertex set with a special source vertex.
  - For each discordant read bicluster, it added a discordant breakpoint edge connecting the respective endpoints of the corresponding sequence edges if both the clusters belonged to the interval set.
  - For all biclusters with one cluster outside the interval set, it introduced a source breakpoint edge connecting the source vertex to the breakpoint vertices corresponding to the clusters within the amplicon intervals.
  - It also added source breakpoint edges connecting the source vertex to breakpoint vertices corresponding to meanshift vertices with no corresponding discordant biclusters and to endpoints of the amplicon intervals.
  - Finally, AA connected breakpoint pairs corresponding to consecutive sequence edges within each interval with concordant breakpoint edges.
  - For each sequence edge and breakpoint edge, AA recorded the number of reads and read pairs respectively mapping to the edge.

A correctly reconstructed breakpoint graph represents a superimposition of all amplicon structures. Each cyclic structure forms a cycle of alternating sequence and breakpoint edges. A linear structure with endpoints connected to genomic positions outside the cycle, can be represented as an alternating closed walk starting and ending at the source vertex. The breakpoint graph construction may not always be complete due to missing edges leading to inaccuracies in final prediction of the structures. This problem was partially alleviated by the fact that, if AA failed to detect a discordant breakpoint edge, but detected the locations of the rearrangement through meanshift boundaries, then the corresponding amplicon structures were represented as one or more walks starting and ending at the source vertex. Henceforth, we restrict the definition of cycle in the breakpoint graph, as a closed walk with alternating sequence edges and breakpoint edges with the exception that the walk may contain two consecutive breakpoint edges connected to the source at most once.

(iv) Copy number estimation using balanced flow optimization: Next, we note that the CN of each edge is the sum of CNs of each amplicon structure where each traversal of the edge is counted separately. As a result, the copy numbers in the graph follow a balanced flow property wherein the CN of a sequence edge matches the sums of CNs of breakpoint edges connected to each breakpoint vertex of the sequence edge. AA modeled the number of read fragments mapping to each edge as a Poisson distribution with the parameters determined by the CN, edge length and sequence coverage parameters. Under this model, AA estimated the copy number CN_seq_ for each sequence edge and CN_bp_ for each breakpoint edge by optimizing a balanced flow (linear constraints) with the convex objective function:^24^
  $$\mathop {\sum }\limits_{\rm seq \in SEQ_G} {\mathrm{\theta }}_{1000} \cdot {\rm CN_{seq}}/R - k_{\rm seq} \cdot \ln \left( {{\mathrm{\theta }}_{1000} \cdot {\rm CN_{seq}}/R} \right) \\ + \mathop {\sum }\limits_{\rm bp \in BP_G} S_{\rm CN_{bp}} - k_{\rm bp} \cdot \ln \left( {S_{\rm CN_{bp}}} \right)$$
  where SEQ_G_ represents all sequence edges and BP_G_ represents all breakpoint edges in breakpoint graph G, *k*_seq_, and *k*_bp_ represent the number of reads mapping to sequence edge seq and the number of read pairs mapping across breakpoint edge bp respectively with the constraint:
  $$\forall v \in \left( {V_{\mathrm G}} \right),\,{\rm CN_{seq_v}} = \mathop {\sum }\limits_{\forall \rm bp|v \in bp \in BP_G} {\rm CN_{bp}}$$
  where seq_v_ represents the sequence edge connected to breakpoint vertex v, *V*_G_ represents set of breakpoint vertices in breakpoint graph G. The optimal solution for the balanced flow was obtained using the convex optimization package Mosek version 8.0.0.60 (https://www.mosek.com). For the third stage output, AA reported the graph edges and their copy counts as text output.
(v) Cycle decomposition: As described above, a linear or cyclic amplicon structure can be represented as one or more cycles in the breakpoint graph. However, even with correct reconstruction and CN assignment to the breakpoint graph, the cycles cannot always be inferred unambiguously, especially with repeated traversals of an edge. Conversely, there may be two or more possible sets of cycles and associated copy numbers, such that combining the cycles within each set may finally result in the same breakpoint graph with the same copy number assignments. Here combination of cycles simply means summing up the copy numbers for each graph edge from each cycle. It is not always practical to enumerate all possible amplicon structures because the number of possible structures can be exponentially large. To address this issue, we first observed that a cycle traversing an edge multiple times in the same direction can be divided into two smaller cycles and conversely, the two cycles can be merged to form the original cycle. However, if we iteratively merge multiple cycles together, then changing the order in which the cycles are merged can produce different resulting structures all with the same edges and copy counts. Based on this observation, AA decomposed the breakpoint graph in to simple cycles, with the aim to represent a large number of amplicon structures using relatively few cycles. We defined a simple cycle as a cycle, which traverses any sequence edge at most once in each direction and hence cannot be divided into smaller cycles. We defined the decomposition of the breakpoint graph as a set of simple cycles with CN assignment such that the CNs of any edges in all the simple cycles sum up to the CN in the breakpoint graph. Although a breakpoint graph may have multiple decompositions and the simple cycles in a single decomposition may not be always be combined to form every possible amplicon structure, these cases require the breakpoint graph to have certain complex patterns expected to occur in a small fraction of amplicons. Instead, AA decomposed the breakpoint graph using a polynomial time heuristic, which iteratively picked the simple cycle with the highest CN and decremented the CN from the corresponding edges in the breakpoint graph. With this algorithm, AA could prioritize the structures that had the highest CN, as well as the cycles, which occur in a large number of structures providing a meaningful way of highlighting the important features of the amplicon. AA provided a text file containing the ordered list of segments within in each simple cycle. Additionally, AA provided an interactive visualization of the simple cycles called the “CycleView”, which could be merged to into larger cycles to investigate possible amplicon structures (Fig. 1h). In the Cycle View, AA displayed the segments aligned with their genomic position in the SV view and consecutive segments were placed on consecutive rows (Figs. 1g, h). If two cycles contained overlapping segments, then a user could select the cycles, their overlapping segments and merge the cycles to form larger cycles. The Cycle View provided a way to interpret the structure of the cycle while visualizing the genomic location and annotations (Figs. 1i, j).

### 3) Samples reported by other studies

We ran AA on previously reported amplicon and provide a comparison of AA reconstructions with previous studies (Supplementary Information). The samples included:

Dataset (i) contained six samples (HL-60, GLC-1-DM, GLC-2, GLC-3, COLO320-DM, and COLO320HSR) provided to us by the authors of the original paper. Each sample was predicted by the original study to contain an amplicon with the oncogene MYC, along with PCR validation of breakpoint edges. We mapped the WGS samples to with coverage between 4.6× and 10.5× and remapped the reads to hg19 reference genome with BWA MEM. We picked the seed intervals using the ReadDepth as described in Methods 1.

Dataset (ii) had three glioblastoma samples (TCGA-06-0648, TCGA-06-0145, and TCGA-06-0152) from Sanborn et al.^20^, also studied by Dzamba et al.^19^. We downsampled the bam files to coverage between 4× − 7× by selecting read pairs with specific read group identifiers. The read group identifiers were selected to be sets of identifiers with the same read length and roughly similar insert length. The exact identifiers selected are mentioned in Supplementary Information. We picked seed intervals based on calls from CNV calling tool ReadDepth with copy number > 5 and size > 100 kbp as described in Methods 1A.

Dataset (iii) consisted of 12 HPV infected cancer samples (HNSCC and CESC) from Akagi et al^28^. For each sample, we predicted the HPV strain as described in Methods 13, remapped all the reads to the combined reference by concatenating the hg19 human reference genome to the reference genome of predicted HPV strain, and used the interval corresponding to the predicted HPV genome as the seed interval.

### 4) Simulation algorithm

We developed a simulation algorithm, AAsim, to simulate 960 amplicons with known “true” structures to measure the accuracy of AA. Simulations of AAsim can be flexibly adjusted through multiple input parameters including: i) interval size, ii) copy number, iii) number of rearrangements, iv) probability of duplication, and v) depth of coverage. To allow testing, the reconstruction without the bias of seed selection, AAsim simulated viral (HPV16) human hybrid structures. The HPV16 genome served as a de facto seed interval such that AA could be tested without providing any additional information about the span of the amplicon. AAsim simulated ecDNA structures through the following steps:

1. Chose a random location on the human genome and integrate the HPV16 genome at this location.
2. Randomly select an interval of input interval size around the site of integration and circularize it to create an ecDNA element containing the human interval with the integrated virus.
3. Iteratively perform rearrangements on the ecDNA, including deletions, duplications, inversions, and translocations such that in each iteration. Each type of rearrangement is selected with preset probabilities, as described below. The breakpoint coordinates for each rearrangement are chosen uniformly at random from the entire ecDNA structure, but rearrangements that deleted the viral genome entirely were not permitted. Iterations were performed until the required number of rearrangements were induced.
4. Record the order of segments in the final structure to be used as “truth” set and assigned a copy count to the ecDNA using the input parameter.
5. Generate 100-bp paired-end reads from the ecDNA using the ART Illumina read simulator^33^ with given depth of coverage. Reads are also generated from other regions of the reference genome for AA to estimate the profile of the sequencing depth.

The output of AAsim included the target “true” amplicon structures and the paired-end reads simulated for these amplicons. The values chosen for the input parameters were: i) Interval size: 40 kbp, 160 kbp, 640 kbp, 2.4 Mbp; ii) Copy number: 4,16, 32; iii) Number of rearrangements: 0, 4, 8, 16, 32; iv) Probability of duplication: 0, 0.25, 0.5, 0.75; v) Depth of coverage: 1, 4, 16, 32. In total, we generated 960 simulations for all combinations of these parameters. The sets of simulations with increasing duplication probability presented test sets with increased difficulty of reconstruction due to larger number of cycles per structures, as well as larger number of segments. To verify that evaluating on simulated episomes alone vs episomes with WGS background does not significantly affect the accuracy estimate, we randomly selected 20 simulated episomes, merged the reads with reads from a simulated tumor genome and compared the number of errors AA in reconstructions of the matched pairs of simulations. In order to estimate the runtime of AA for larger amplicons, we simulated 24 more structures and reads with parameters: i) Interval size: 5 Mbp, 10 Mbp; ii) Copy number: 32; iii) Number of rearrangements: 0, 8, 32; iv) Probability of duplication: 0, 0.25, 0.5, 0.75; v) Depth of coverage: 32. To test for change in accuracy with copy number and coverage, we downsampled the reads to i) Copy number: 4,16, 32; ii) Depth of coverage: 1, 4, 16, 32.

### 5) Measuring accuracy of AA

A) Accuracy of methods for SV and CNV analysis

SV and CNV analyses provide the building blocks for AA to accurately reconstruct the breakpoint graph and ultimately predict the full structure. In order to establish the reliability of these critical components, we measured the accuracy of methods for interval detection, discordant edge detection, and meanshift edge detection. In terms of interval detection, even though AAsim simulated an ecDNA circularized from a single interval, the final structure can have multiple intervals due to deletion of intermediate segments. We measured the number of intervals completely identified by AA (TP), the number of intervals not completely identified by AA (FN), as well as the number intervals reported by AA, which did not have any amplification (FP). For measuring the accuracy of CNV detection, we measured the number of CNV boundaries correctly (within 10 kbp) identified by the meanshift edge detection algorithm (TP), the number of CNV boundaries not identified (FN), as well as the number of locations reported which actually did not have a copy number change (FP). Similarly, for discordant edges, we reported the number of edges with both breakpoints predicted correctly (within 300 bp) (TP), one or more breakpoints not detected (FN) and discordant edges reported which did not exist in the true structure (FP) (Supplementary Fig. 7a-c).

B) Edit distance computation and reporting

For measuring the accuracy of final amplicon reconstruction, we defined a distance measure to quantify the difference between the predicted structure as compared with the true structure from the simulation. Inspired by the genome sorting problem, the goal of the distance measure is to represent the number of operations to transform the predicted structure into the true structure. The genome sorting problem aims to find the distance between two related genomes by counting the minimum number of operations to transform one genome into another. These operations may include inversions^32^, translocations^34^, or double-cut-and-join (DCJ) operations^35^. We adapted the DCJ operation to measure reconstruction accuracy by defining the Repeat-DCJ (RDCJ) distance, which can separately count the portion of operations caused due to reconstruction errors and due to alternative traversals across repeats (Supplementary Fig. 6). The RDCJ distance is defined as the sum of a two-part measure: i) repeat branch swaps and ii) reconstruction errors. First, the prediction may have errors caused by inaccurate breakpoint graph construction including missing or false breakpoint edges and inaccurate copy numbers of segments. We denote these as reconstruction errors as they represent errors caused by the reconstruction algorithm. Reconstruction errors involve addition and deletion of breakpoint edges. On the other hand, under perfect graph construction, the only operation needed to transform the predicted structure into the true structure is to transform the order of traversal across repeated segments without any change in the set of breakpoint edges used. We call this operation a repeat branch swap. Two cycles can be merged together through a single repeat branch swap. In measuring the RDCJ distance, we simultaneously minimize the number of reconstruction error corrections and repeat branch swaps required for the transformation.

Under the RDCJ model, we count the edits on each segment independently and sum these up to obtain the total distance for the entire reconstruction. To achieve this, we represent each segment by a switch. A switch is a bipartite graph where the two parts represent the start and end of the segment, respectively, and the vertices are the union of breakpoint vertices from the true and predicted structures. If a breakpoint vertex is traversed multiple times in either of the structures, then we create multiple copies of the vertex equal to the maximum number of traversals in the two structures. We define a switch graph as a graph, which consists of all the switches as subgraphs connected through connective edges, which are union of the breakpoint edges from the breakpoint graphs of the true and predicted structures with appropriate multiplicities. Each switch vertex has exactly one connective edge. Finally, each of the true and predicted structures form a walk on the switch graph inducing respective matchings within the bipartite switches. The edges within the matching, called match edges, connect consecutive breakpoint edges in the structure. As a result, we represent the edit distance of the predicted structure to the true structure by counting the number of operations of transforming each switch independently.

Consider a switch with vertices *V*_1_, *V*_3_ on one shore, and *V*_2_, *V*_4_ on the other shore of the bipartite graph with switch edges (*V*_1_, *V*_2_) and (*V*_3_, *V*_4_). In using a repeat branch swap to transform the predicted matching to the true one, we can, for example, replace edges (*V*_1_, *V*_2_) and (*V*_3_, *V*_4_) by two new edges (*V*_1_, *V*_4_) and (*V*_3_, *V*_2_). Note that a repeat branch swap represents a copy number neutral operation and correspondingly, the number of switch edges does not change. As the set of vertices in the matching is invariant, the set of connective edges connected to matched vertices also does not change. In contrast, correction of reconstruction errors involves addition, deletion or reassignment of exactly one vertex of a match edge.

To measure the accuracy of AA, we counted and reported the required the number of the operations of both types across all switches for each simulated structure. We classified the 960 simulated structures into four groups based on the value of the parameter probability of duplication, representing amplicons, which are increasingly difficult to reconstruct. To provide a standard yardstick, we compared the reconstruction errors of AA predictions against those of a random predictor. The random amplicon structure consisted of a randomly shuffled order of all segments from the true amplicon structure. Notably, the average performance of the random predictor closely followed two times number of segments in the amplicon where number of segments is measured by adding up counts of segments. Based on this observation, we defined the error rate of one or more predictions as the total reconstruction errors as a percentage of 2 × total number of segments (Figs. 1k–n, Supplementary Fig. 7d-g).

### 6) Runtime computation

We recorded the runtime of AA for each simulated amplicon using the Python function time.time(). We plotted a scatter plot of the runtime as a function of the total DNA content of the amplicon on a logscale graph to capture performance on small and large amplicons (Supplementary Fig. 8b). The total DNA content was defined as length of ecDNA structure × copy number × depth of coverage. We plotted the best fit line on the logscale graph for the runtime as function of the total DNA content using the linregress function available in the Python library scipy.stats.

### 7) SRA samples, ReadDepth CNV calls

We used sequencing data from 117 cancer samples including cell lines, patient-derived xenografts (PDXs) and tissue samples and eight normal control samples originally described in Turner et al.^9^. These samples may be downloaded from NCBI Sequence Read Archive (SRA) under Bioproject (accession number: PRJNA338012). Additionally, we studied WGS of 18 biological replicates of seven samples totaling to 135 cancer WGS datasets. Four of these samples had replicates treated with targeted drugs and glioblastoma PDX GBM39 also had post-treatment replicates (Supplementary Data 1). FISH results for oncogene probes reported in Turner et al. were used to mark amplicons to be present on EC only, HSR only or both EC and HSR (Fig. 2d, Supplementary Data 3).

After reconstruction of amplicons in 117 WGS tumor samples using the 255 seed intervals from ReadDepth, AA reconstructed 135 amplicons, which consisted of 265 intervals. Although AA merged multiple seed intervals into larger intervals, AA reported 63 new intervals not intersecting the seed intervals including possible false positives from repetitive regions (Supplementary Data 2,3).

### 8) TCGA interval set and somatic CNV identification

We downloaded 22,376 masked CNV call files from TCGA^25^ generated from Affymetrix 6.0 data for 10,995 cases. We mapped the original calls from hg38 coordinates to hg19 coordinates using the Liftover tool from UCSC genome browser. We selected 10,494 cases for which Liftover successfully mapped the calls for at least one cancer sample and one matched normal sample. For cases with multiple call sets, we took the average copy number of each segment for cancer and normal call sets, respectively. Next, we selected the CNV calls in the tumor samples according to the seed interval selection procedure described in Methods 1. However, we did not exclude the calls from the blacklisted regions since we believe those regions only need to be blacklisted due to artifacts specific to WGS samples and not array data. Using this method, we obtained amplified intervals in 2527 cancer cases. Comparing the copy number calls in cancer samples with copy numbers in matched normal samples, we filtered out calls where the difference in copy number was <3 (specifically, copy number at least 5 when there was no CNV call in the normal sample). This criterion only filtered out a small number of intervals resulting in 12,162 intervals in 2513 cancer cases. We used this set for further analysis.

### 9) Overlap of AA amplicons with intervals amplified in TCGA

We tested whether amplicons reconstructed by AA in sample set 1 were representative of the focal amplifications across human cancer by testing whether the overlap between AA amplicons with TCGA intervals was significantly larger than expected by random change. As originally described in Turner et al.^9^, for each sample, we computed a match score between the AA amplicons for the sample and the TCGA intervals from the corresponding cancer types, which were amplified with frequency > 1%. The match score for the sample was simply the sum of frequencies of the TCGA intervals within the corresponding cancer types that overlapped an amplicon from the sample. We recorded the cumulative match score as the sum of match scores for all samples in sample set 1.

To test if the cumulative match score for the TCGA intervals was significantly larger than expected by random chance, we generated 720 million permutations of the TCGA intervals, which were amplified with frequency > 1% in each cancer type by assigning random positions to the intervals within the human reference genome while maintaining their size. We computed the cumulative match score of each permutation with sample set 1 using the same procedure as above and found that eight permutations had a larger match score than the original TCGA intervals. The *p*-value of the significance of overlap between amplicons in sample set 1 and TCGA intervals was reported as 8/720 million = 1.1×10^−8^.

### 10) Size and copy number determination and exponential distribution

The size of an amplified interval was defined as the sum of sizes of all amplified segments with copy number > 5 within the interval. The size of the amplicon was the sum of sizes of all intervals in the amplicon. The copy number of an interval was the average copy of the amplified segments in the interval weighted by their size. Similarly, the average copy number of an amplicon was the weighted average of amplified segments in all intervals of the amplicon weighted by their size. In the analysis of final reconstructions, we used the copy numbers assigned to sequence edges by AA rather than CNV calls from ReadDepth. We plotted the scatter plot for copy number vs size of the 135 AA amplicons for the 117 samples and the TCGA intervals (Fig. 2d). For direct comparison, we also plotted the copy number vs size of the AA intervals (Supplementary Fig. 10). We observed six AA amplicons and three AA intervals with sizes > 3 Mbp and copy number > 30, whereas we observed exactly one TCGA interval satisfying these constraints. Particularly, the CNV array calls were capped off at copy number around 40. We verified that this was the case for the amplicons from all four TCGA WGS sample from previous publications where the sequencing data reported higher copy counts.

Next, we plotted histograms for the copy number and size of the TCGA intervals with bin sizes of copy number 1 and size 400 kbp where the height of the histogram was log scaled. (Fig. 2d). Both histograms displayed a linear decay indicating exponential distributions. We obtained the best fit lines for these histograms using the polyfit function in the Python package Numpy based on 20 bins each for copy number (5–25) and sizes (0 bp–8 Mbp) beyond which the data became too sparse. Estimates for the means of the exponential distributions, 3.16 copies and 1.74 Mbp, were obtained using the negative inverse of the slope of the best fit lines in both cases.

### 11) Determination of multi-interval and multi-chromosomal amplicons

We compared the sizes of amplicons containing a single genomic interval to sizes of amplicons with multiple intervals from one or more chromosomes. To be more conservative in the interval selection and avoid falsely detected interval from repetitive regions, we selected intervals based on segments reported to be amplified by the meanshift based CNV analysis. As a result, only high confidence intervals detected by the CNV analysis at a resolution of 10 kbp were selected. Next, for a uniform definition of interval, only for this analysis, we merged all CNV segments within 5 Mbp of a neighboring segment into a single interval. After merging these segments, we classified the amplicons into three categories: (i) Clustered: 104 amplicons containing a single interval, (ii) MultiCluster: 14 amplicons containing multiple intervals from the same chromosome, and (iii) MultiChrom: 17 amplicons containing multiple intervals from multiple chromosomes (Fig. 2f).

We plotted the distribution of amplicon sizes of the entire amplicon for each of the three categories, as well as the size distribution of the set of all intervals in all amplicons in the MultiCluster and MultiChrom categories using the Python Seaborn library function “violinplot”. We compared the sizes of all amplicons and intervals in the MultiCluster and MultiChrom category to the sizes of all Clustered amplicons using Rank-Sum test available through Python Scipy.Stats library function “ranksums”. We observed that the MultiCluster (*p* = 1.58×10^−2^, mean = 4.7 Mbp) and MultiChrom (*p* = 6.18×10^−4^, mean = 7.7 Mbp) amplicons were significantly larger than the Clustered amplicons (mean = 2.3 Mbp). However, the sizes of intervals from all amplicons in the MultiCluster (mean = 2.2 Mbp) and MultiChrom (mean = 2.7 Mbp) categories did not show any significant difference from the Clustered amplicons.

### 12) TCGA subtype-specific enrichment

We considered the significance that the amplicons amplified specifically amplified oncogenes in a tumor-type-specific fashion. To compute the significance, we assume under the null model that each amplicon interval is randomly positioned. For a genome of length *G*, the probability that amplicon *a* of length *l*_*a*_ intersects with oncogene *g* of length *l*_*g*_ is *A*_(*a*,*g*)_ = (*l*_*a*_ + *l*_*g*_)/*G*. Under the approximate assumption that all amplified intervals are independent of each other, the probability that at least one amplified interval in sample *s* randomly intersect *g* is $B_{\left( {s,g} \right)} = 1 - \mathop {\prod }\limits_{a \in s} (1 - A_{\left( {s,g} \right)})$. As each sample is independent, the number of samples with an amplicon intersecting a particular oncogene follows a Poisson Binomial distribution with *n* trials where *n* is the number of samples with success probabilities *B*_(1, *g*)_, *…, B*_(*n*,*g*)_. Thus, if for each tumor type with *n* samples, *g* is amplified in *k* samples, then we calculated the *p*-value for this observation under the null model using the PoiBin function provided by https://github.com/tsakim/poibin. To answer whether amplifications of *g* are significantly enriched in the given tumor type, we recorded an enrichment if the Bonferroni-corrected *p*-value was < 0.05, using correction factor *G*/*l*_*g*_ (Fig. 2g).

### 13) Similarity of amplicons overlapping an oncogene

To investigate whether amplicons containing an oncogene also contained other genomic elements that played a significant role in the formation or amplification process, we measured the similarity between amplicons from different samples containing one of the oncogenes *EGFR*, *MYC*, and *ERBB2* from the WGS dataset of 117 samples. For a given oncogene, if additional genomic elements played a significant role, then we would expect a larger overlap within amplicons from multiple samples containing the oncogene than by random chance. We measured the pairwise similarity between amplicons from two samples containing an oncogene, we calculated the size of the overlap between the amplicons and quantified the significance of the size of the overlap with respect to a null distribution for the size of the overlap. The null distribution was set as the distribution of size of overlap for all valid configurations for both amplicons, where a valid configuration of an amplicon was defined as any assignment of a chromosomal location for the start position of the first interval such that all the intervals maintained their order and sizes as the observed amplicon, as well as the distances between consecutive intervals such that at least one interval contains the given oncogene. Given a pair of amplicons, we estimated the null distribution by computing the overlap for all valid configurations obtained by shifting the amplicon intervals from the smallest to the largest possible genomic location in steps of 10,000 bp. Finally, for all amplicons containing each of the three oncogenes, we measured the significance of all pairwise similarities and visualized these through three QQ plots (Supplementary Fig. 11). Through the QQ plots, we observed that in our sample set, that there was significant similarity within amplicons from multiple samples for any of the oncogenes. This suggests that there was no single element that played a significant role in the formation or amplification process.

### 14) Description of cervical cancer samples

We downloaded the sequencing data for 68 cervical cancer samples with matched normal samples from TCGA^25^. We downloaded 337 HPV reference genomes from PapillomaVirus Episteme database (PaVE)^36^ on 15 August 2016 and concatenated these with the hg19 reference chromosomes to create an hg19_hpv337 reference genome. For each of the samples, we randomly extracted 30 million read pairs using the HTSlib bamshuf + bam2fastq utilities and aligned these to the hg19_hpv337 reference genome using BWA mem. We determined the existence and strain of HPV infecting each sample by identifying the strain with the highest number of mapped reads. For each of the identified strains, we separately concatenated the genome of the single strain to the hg19 chromosomes, to create the sample-specific reference genome, for example, hg19_hpv16 and mapped all the reads in the sample to this reference genome using BWA-mem. Finally, for each sample, we ran AA with the --sensitive-ms (Methods 2) option on the mapped reads with the reference genome of the specific HPV strain as the seed interval. From the reconstructions, we combined chromosomal intervals within 5 Mbp and if AA identified multiple amplified intervals connected to the HPV genome, they were treated as different amplicons unless connected to each other through discordant edges. We selected the “amplicons” for which the weighted average of the copy numbers of all amplified (CN > 2.5) sequence edges from the human chromosomes was >3.0. We plotted a scatter plot for the copy number vs size of all amplicons similar to Fig. 2e (Supplementary Fig. 12) and observed that virus-induced amplicons had a mean size of 155 kbp and mean copy number of 7.04.

### 15) Identification of unifocal and bifocal signature

We developed criteria for calling a unifocal and bifocal signature and for each “amplicon” identified according to Methods 14, we manually searched for unifocal and bifocal signatures. An amplicon was defined to contain a unifocal signature if we found two reciprocal discordant edges connecting the virus genome to the human genome such that these edges had opposite strand on each of the genomes and the positions of the edges on the human genome were within 1 kbp of each other. An amplicon was defined to contain a “strong” bifocal signature if it contained a pair of chimeric edges with opposite orientations, which flanked the entire amplified region on the human genome. Otherwise, an amplicon was defined to contain a “weak” bifocal signature if it contained a pair of chimeric edges with opposite orientations such that all the segments within the region flanked by these edges had higher copy number than all the other segments.

### 16) Simulation of unifocal and bifocal integrations

We hypothesized that the formation of amplicons with a unifocal signature was initiated by a unifocal integration of the virus into the human genome, whereas a bifocal integration is a more likely mechanism for the formation of amplicons with a bifocal signature. To test this hypothesis, we simulated four sets of amplicons where each set consisted for 40 simulations. Out of the four sets, two consisted of amplicons originating from unifocal integrations and the other two sets consisted of amplicon originating from bifocal integrations. For the two types of integration, we simulated one set each of linear chromosomal amplicons and circular extrachromosomal amplicons. Thus, if four-ordered segments ABCD represent a section of the normal human genome and V represents a viral segment, then the circular and linear amplicons with unifocal integration had the initial structures (BVC) and BVC, respectively. The circular and linear amplicons with bifocal integration had the initial structures (BV) and BVB, respectively. Here “()” represents a circular structure. For each set, the length of an amplicon, B + C in case of amplicons with unifocal integration and B in case of the amplicons with bifocal integrations, was chosen from an exponential distribution with mean 155 kbp matching the mean of the amplicons detected in the 68 cervical cancer samples. In case of the unifocal distribution, the location of integration defining the segments B and C was chosen uniformly through the amplicon interval. For each simulation in all the four sets, we iteratively performed 20 rearrangements, which was comparable to the maximum number of rearrangements in our sample set. The type of each rearrangement was chosen randomly from the set: {translocated duplication, tandem duplication, inverted duplication, translocation, inversion, deletion} with probabilities: {0.19, 0.19, 0.19, 0.19, 0.19, 0.05}, respectively and the coordinates for each rearrangement were chosen uniformly randomly from the amplicon structure formed after the previous iteration of rearrangement with the constraint that the rearrangement may not delete all segments from the viral genome. The probability of deletion was lower than the other rearrangements to make sure that too many deletions did not remove large chunks from the amplicon. After each iteration, we tested whether the amplicon structure could show a unifocal signature based on the existence of a pair of proximal viral connections to opposite strand of the human genome within 1 kbp of each other. Similarly, we tested if the amplicon structure could show bifocal signature by checking if the virus had connection flanking the outmost ends of the human segments in the amplicon. For each set of 40 simulations and after each iteration of rearrangements, we reported the number of amplicons that showed a unifocal signature and the number of amplicons that showed a bifocal signature (Supplementary Fig. 13). We found high fidelity between the type of integration and the observed signature in the amplicon. To elaborate, we found that most amplicons with unifocal and bifocal integrations showed unifocal and bifocal signatures respectively, but it was rare to observe amplicons with a unifocal integration to show a bifocal signature or amplicons with a bifocal integration to show a unifocal signature. This suggests that amplicons with bifocal signatures were unlikely to have originated from a unifocal integration of the virus, whereas amplicons with a unifocal signature were highly likely to have originated from a unifocal integration.

### Code availability

The AmpliconArchitect software described in the manuscript is available at https://github.com/virajbdeshpande/AmpliconArchitect.

## Supplementary Information

Supplementary Information

Description of Additional Supplementary Files

Supplementary Data 1

Supplementary Data 2

Supplementary Data 3

Supplementary Data 4

Supplementary Data 5

Supplementary Data 6

Supplementary Data 7

Supplementary Data 8

Source Data

## Data Availability

WGS data for sample set 1 and six replicates for sample GBM39 were downloaded from the NCBI Sequence Read Archive (SRA) under BioProject (accession number: PRJNA338012). Twelve replicates for other samples are uploaded on SRA under BioProject (accession number: PRJNA437014). Source data for the figures is provided in a Source Data file. The reconstructions described in this manuscript may be downloaded from https://figshare.com/articles/AmpliconArchitect_reconstructions/5950339 including (a) all reconstructions on previously reported amplicons, (b) all reconstructions from sample set 1 and replicates, (c) all examples of simple cycles, (d) all multi-interval amplicons, (e) all multi-chromosomal amplicons, (f) sample with Breakage-Fusion-Bridge signature and (g) all reconstructions from sample set 2. All other relevant data are available upon request.
