# Supplementary material for: Exploring the landscape of focal amplifications in cancer using AmpliconArchitect
Source: Nat Commun. 2019 Jan 23;10:392. doi: 10.1038/s41467-018-08200-y (PMC6344493; doi:10.1038/s41467-018-08200-y)
Supplement: Supplementary file 1 — Supplementary Information [file 41467_2018_8200_MOESM1_ESM.pdf]

Supplementary Material

Exploring the landscape of focal amplifications in cancer using AmpliconArchitect

Deshpande et al.

Contents:

- Supplementary Figures 1-14
- Section 1: Performance of AA on previously reported amplicons

Supplementary Figures:

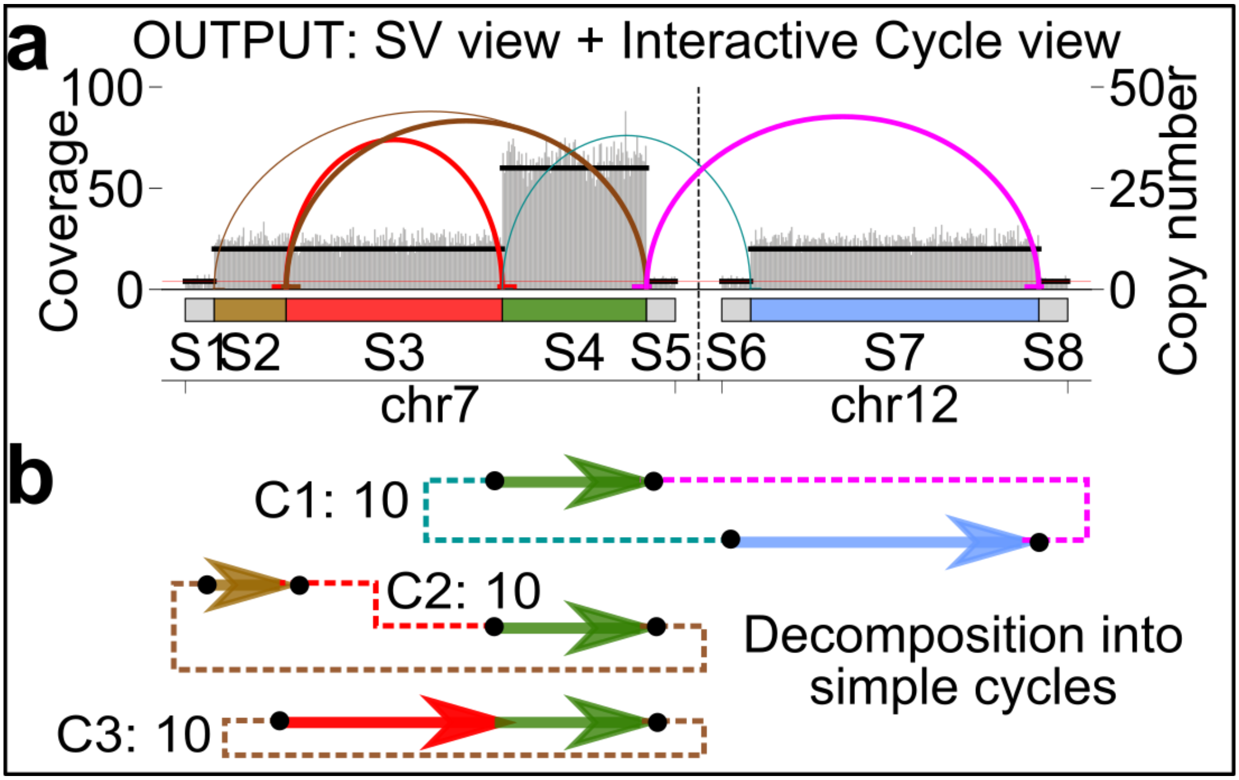

Figure S1: AA visualizations of reconstructed amplicons: (a) The SV view displays multiple data modalities: (i) x-axis shows the set of intervals in the amplicon, (ii) grey histogram and scale on the left y-axis show the depth of coverage through the intervals, (iii) horizontal black lines and scale on the right y-axis show the predicted segmentation and initial CN estimate based on the meanshift technique, (iv) arcs showing discordant edges are color coded for read mapping orientation – red: length discordant, brown: everted, teal: forward, magenta: reverse, blue: outside amplicon (not shown) and (v) bottom panel in (a) shows sequence edges determined based on the SV and CNV signatures and their labels. Note, that in the actual SV view generated by AA, the segments in the bottom panel are replaced by oncogene annotations, e.g. Figure 2a, b. (b) The cycle view shows the simple cycles predicted.: (i) arrows are the sequence edges (contiguous segment that form the ecDNA structure). They are aligned vertically with their position in 1G and match their colors with the annotation in the SV view, (ii) dashed lines represent breakpoint edges and match the color of the corresponding arc in the SV view. Labels show cycle ID and predicted copy number. Note, in the actual web interface, the arrows are replaced by uncolored rectangles and dashed lines are replaced by solid lines, e.g. Figure 2c.

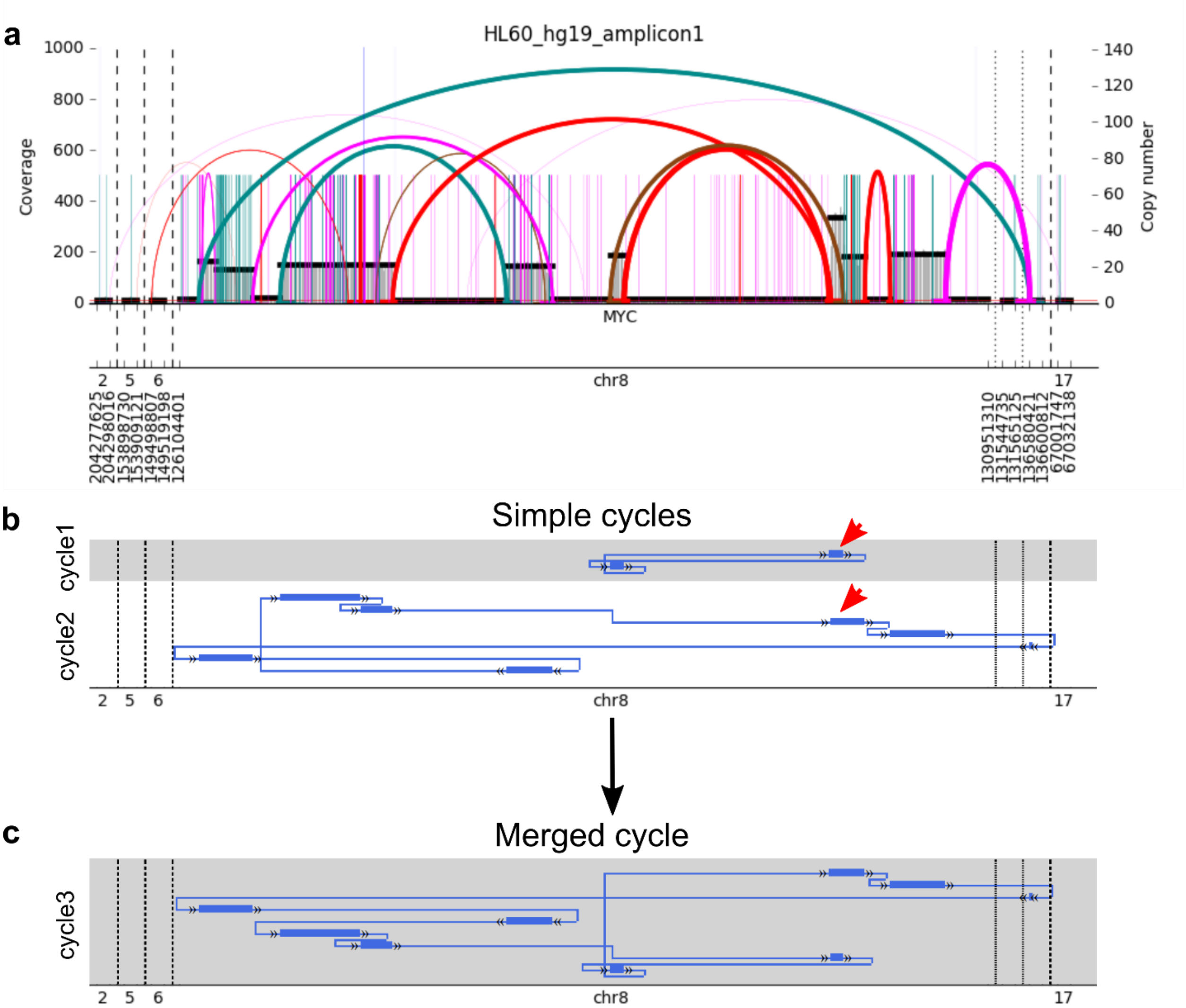

Figure S2: Example of the cycle merging operation: (a) SV View of amplicon containing oncogene *MYC* in leukemia cell line HL-60, (b) Cycle View of top 2 simple cycles (cycle1 and cycle2) reconstructed by AA. Red arrows point to overlapping segments used to merge the two cycles, and (c) A large amplicon structure (cycle3) obtained by merging cycle1 and cycle2 merged using the overlapping segments.

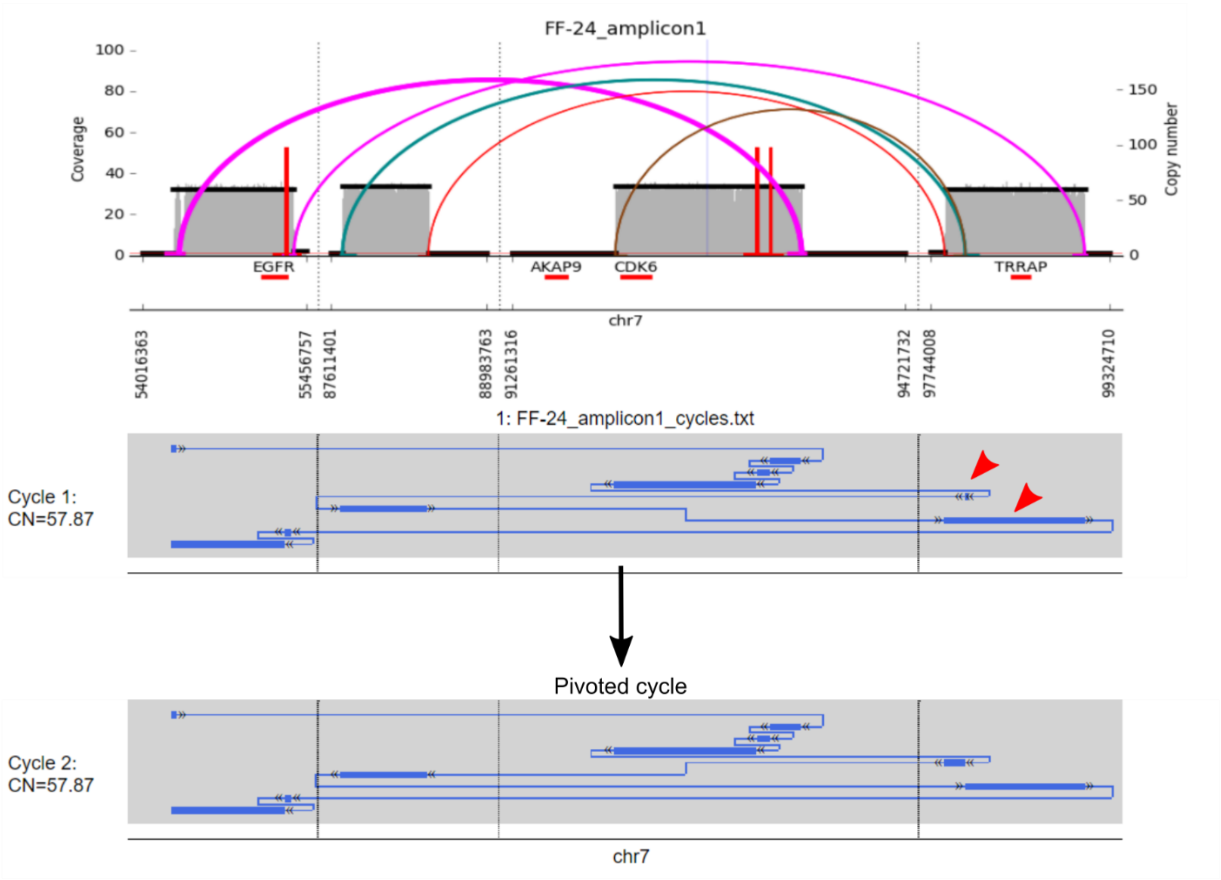

Figure S3. Example of the cycle pivoting operation: Top: SV View of amplicon containing oncogenes *EGFR*, *CDK6* and *TRRAP* in glioblastoma sample FF-24; middle: Cycle View of the top simple cycle reconstructed by AA (Cycle 1). Red arrows point to overlapping segments within the cycles, and bottom: a cycle (Cycle 2) obtained by pivoting cycle 1 by reversing the traversal order of the segments between the 2 read arrows.

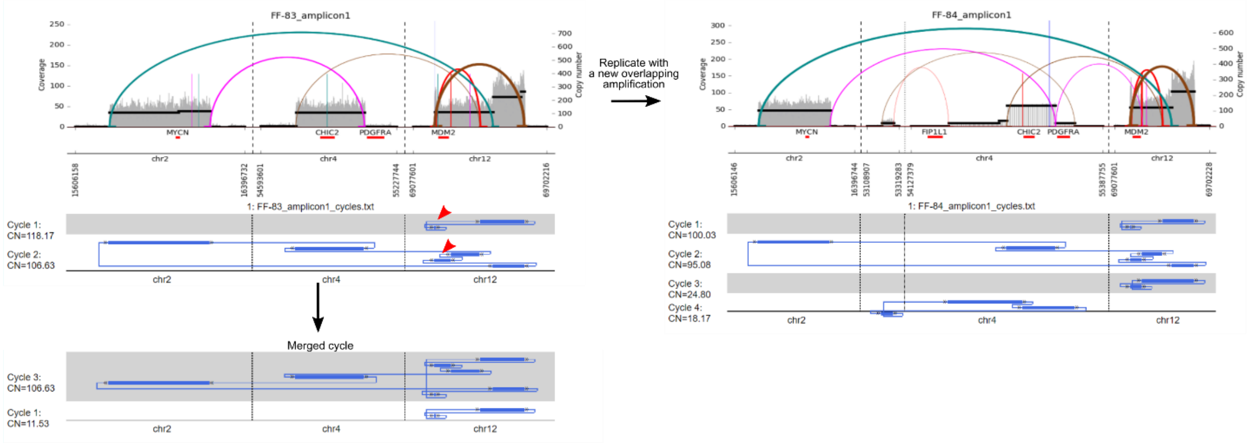

Figure S4. Example of the amplicon evolution predicted by AA: Left panel shows a GBM cell line with a complex amplicon consisting of 2 simple cycles (Cycle 1 and Cycle 2) which can be

merged into a single large cycle (Cycle 3). Red arrows point to overlapping segments used to merge the two cycles. Right panel shows a replicate which acquired a new amplification (Cycle 4) overlapping with the original amplicon resulting in a heterogenous mixture of 2+ structures with different copy numbers. AA is able to reconstruct the structure of the new amplification independently of the original structure.

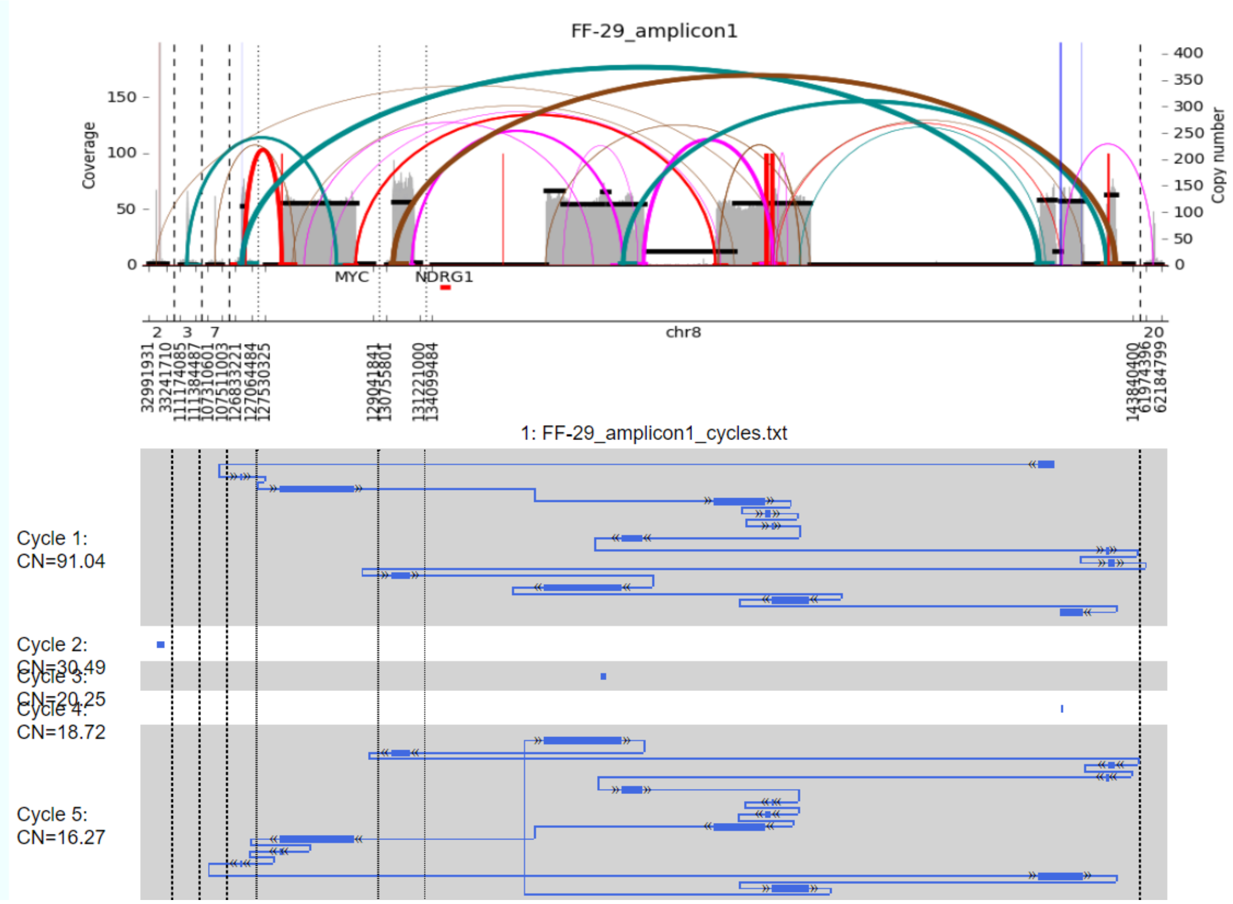

Figure S5. AA reconstruction of complex amplicon with multiple breakpoint edges. SV view and cycle view of *MYC* amplicon in a medulloblastoma cell line. The top cycle (Cycle 1) in AA predicts a structure with 13 segments where the connection of the first and last segment is missing due to an undetected breakpoint edge. The amplicon also contains other rearrangements with low copy number relative to the average copy number of the amplicon. These are filtered by the cycle reconstruction algorithm of AA in order to distinguish the dominant structure.

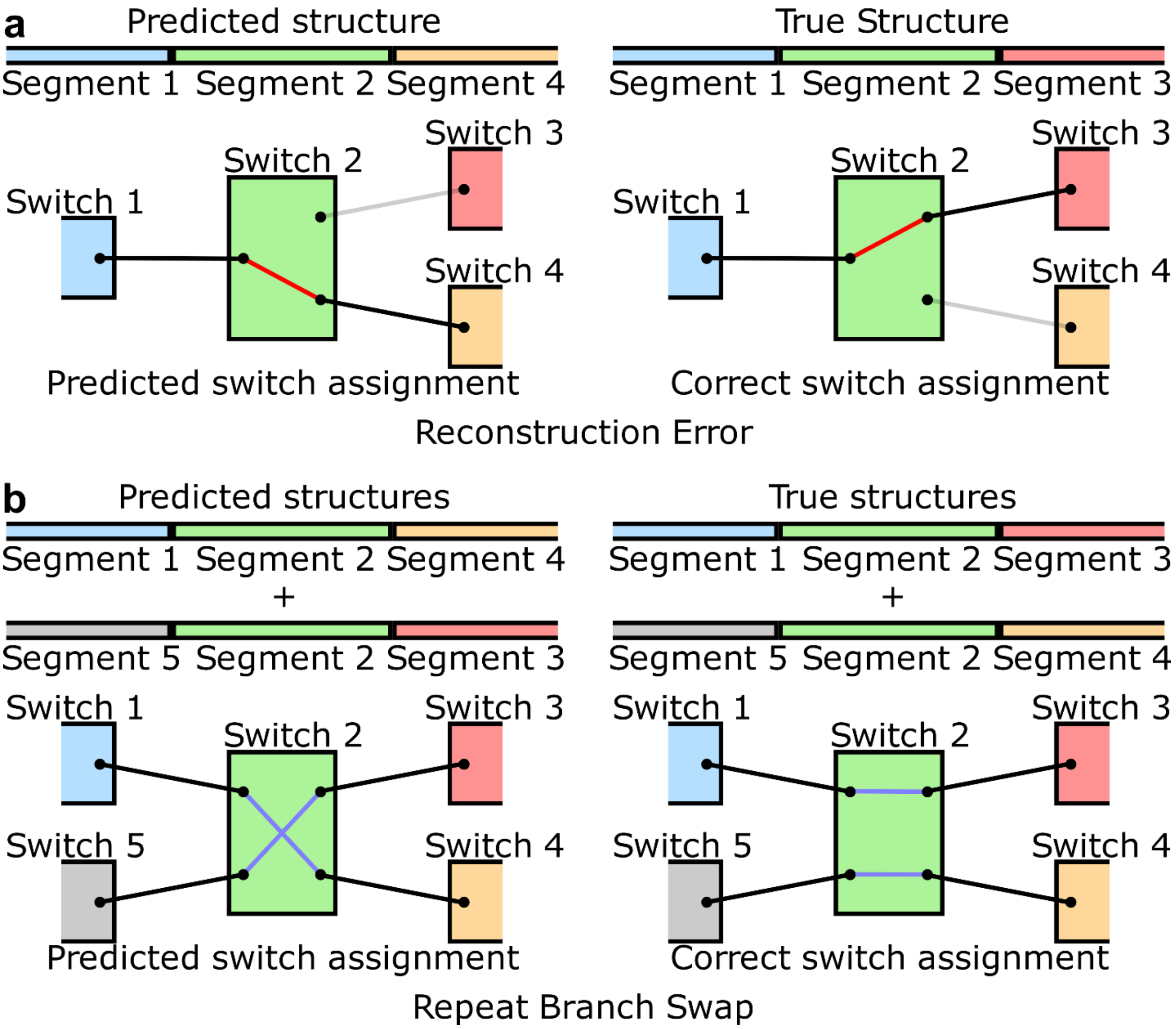

Figure S6: Error model for benchmarking AA: The error model quantifies edit distance of predicted structure from the true structure in terms of the number of graph operations required to transform predicted structure into the true structure. Each segment in either structure is represented by a 'Switch' which is a bipartite graph. Each shore of the bipartite switch represents connections on one side of the segment to neighboring segments through breakpoint edges (black). Each bipartite edge corresponds to one copy of the segment in an amplicon structure. Graph operations are divided into 2 categories: (a) Reconstruction errors: operations involving changes in copy numbers of segments or breakpoint edges due to errors in SV analysis by AA and (b) Repeat branch swaps: operations which change the order of segments in the amplicon structure without changing the copy number of any segment or inter-segment connections. A cycle merging operation is achieved through a repeat branch swap.

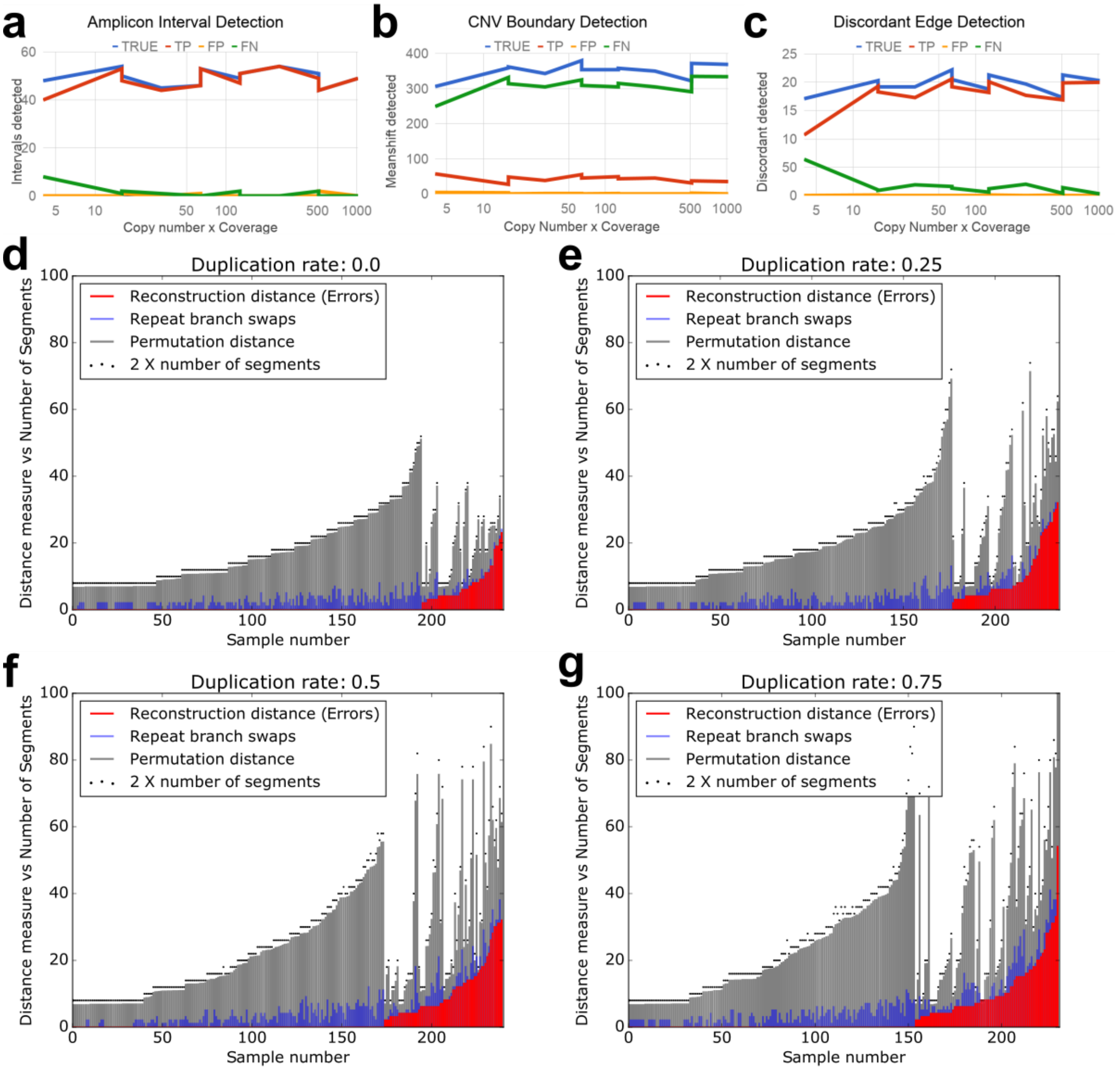

Figure S7: Accuracy of AA submodules and reconstruction: Detection accuracy of AA submodules for 3 amplicon features: (a) Amplicon intervals, (b) CNV boundaries and (c) Discordant breakpoint edges showing the TRUE (blue) count of the feature in simulated amplicons, the number of correctly detected features (TP, red), number of undetected features (FN, green) and the number of incorrectly detected features (FP, orange). The precision/recall of AA for the 3 measures was as follows: (i) Detection of amplified intervals - 99.3%/97.3%, (ii) Detection of copy number shifts - 97.7%/12% and (iii) Detection of discordant breakpoint edges - 99.9%/92%. Low sensitivity of copy number shifts is expected because we included small amplicons starting at 40kbp with up to 16 rearrangements as compared to a detection window size of 10kbp. This has limited effect on final reconstruction since the exact copy number shifts are mostly useful when breakpoint edges are not detected. If discordant reads are present, we can define the breakpoint with high precision. (d-g) Number of reconstruction errors (red bars) and repeat branch swaps (blue bars) for each simulated amplicon structure contrasted with the average number of errors in randomly predicted structures by the naïve 'permutation predictor'

(grey bars) and 2 × the number of segments in the amplicon structure (black dots). Simulated structures grouped by duplication rate for the simulation.

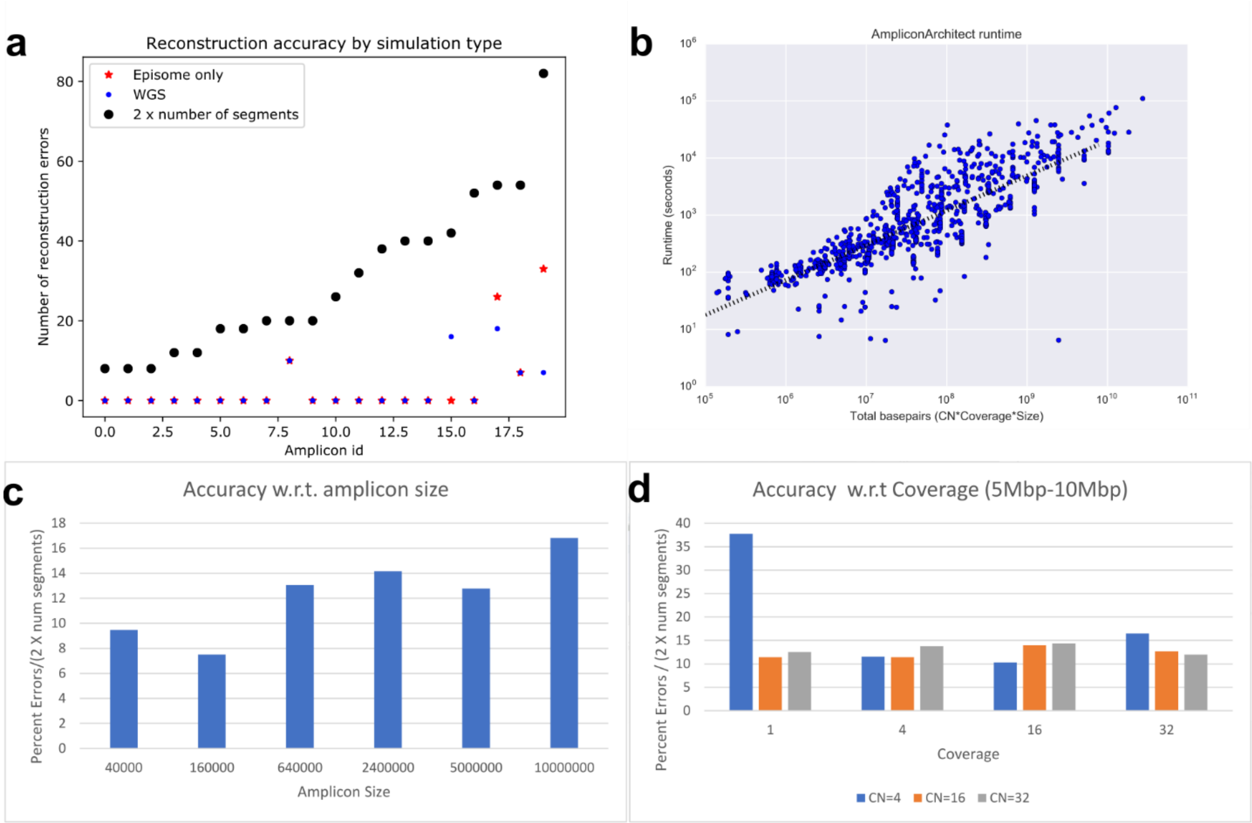

Figure S8. Accuracy and runtime of AA with changing parameters: (a) Number of errors in AA reconstructions for samples with reads simulated from episome only (red stars), vs reads from WGS for simulated sample with amplicon. Samples shown include 20 randomly chosen samples out of the set of 960 simulations described in Methods 4. (b) Computational runtime of AA for each simulated amplicon as a function of total number of basepairs in whole genome sequencing data associated with amplicon. The total number of base-pairs is measured as the size of amplicon structure (bp) × copy number of the structure × haploid sequencing coverage of the sample. (c) Accuracy as a function of amplicon size, where the number of errors and segments is summed over all amplicons in the bin (240 amplicons in the first 4 bins each and 144 amplicons in the last 2 bins each). (d) Accuracy as a function of coverage and copy number, for amplicons of size 5Mbp-10Mbp (24 per bin) where the set of underlying structures is the same for each bin and the number of errors and segments is summed over all amplicons in the bin.

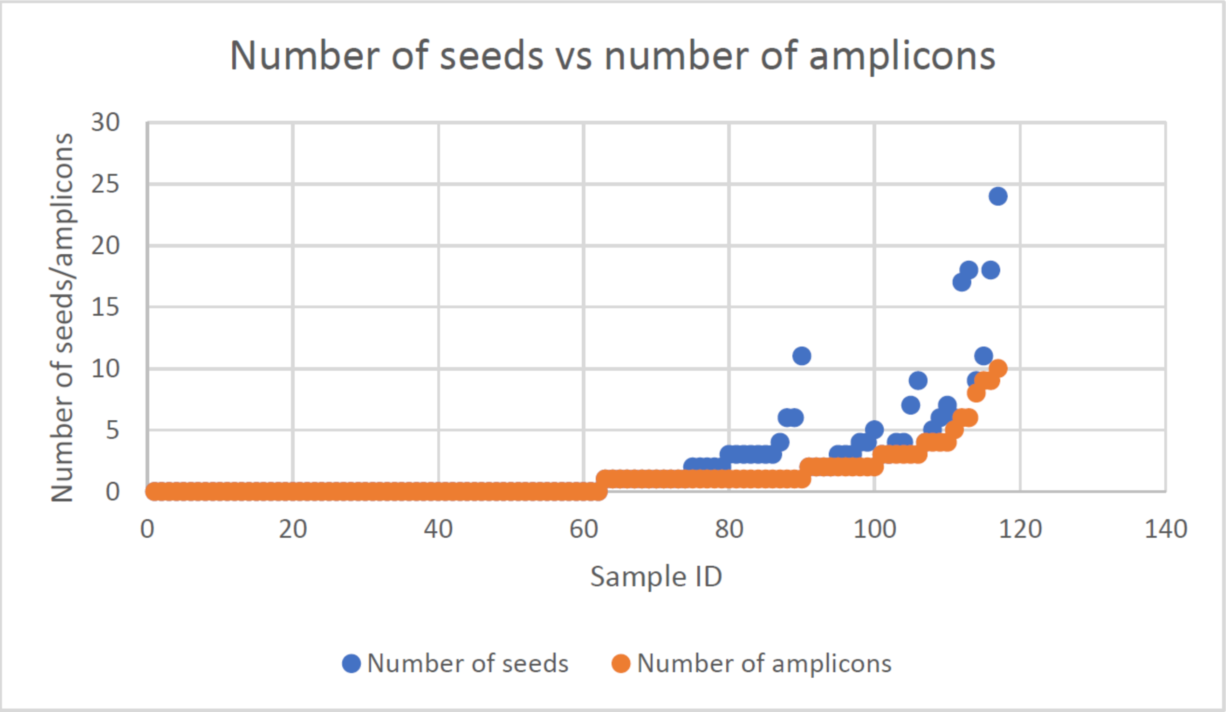

Figure S9. Number amplicons vs number of seeds per sample: Plot shows number of seed intervals and number of amplicons for each sample in sample set 1.

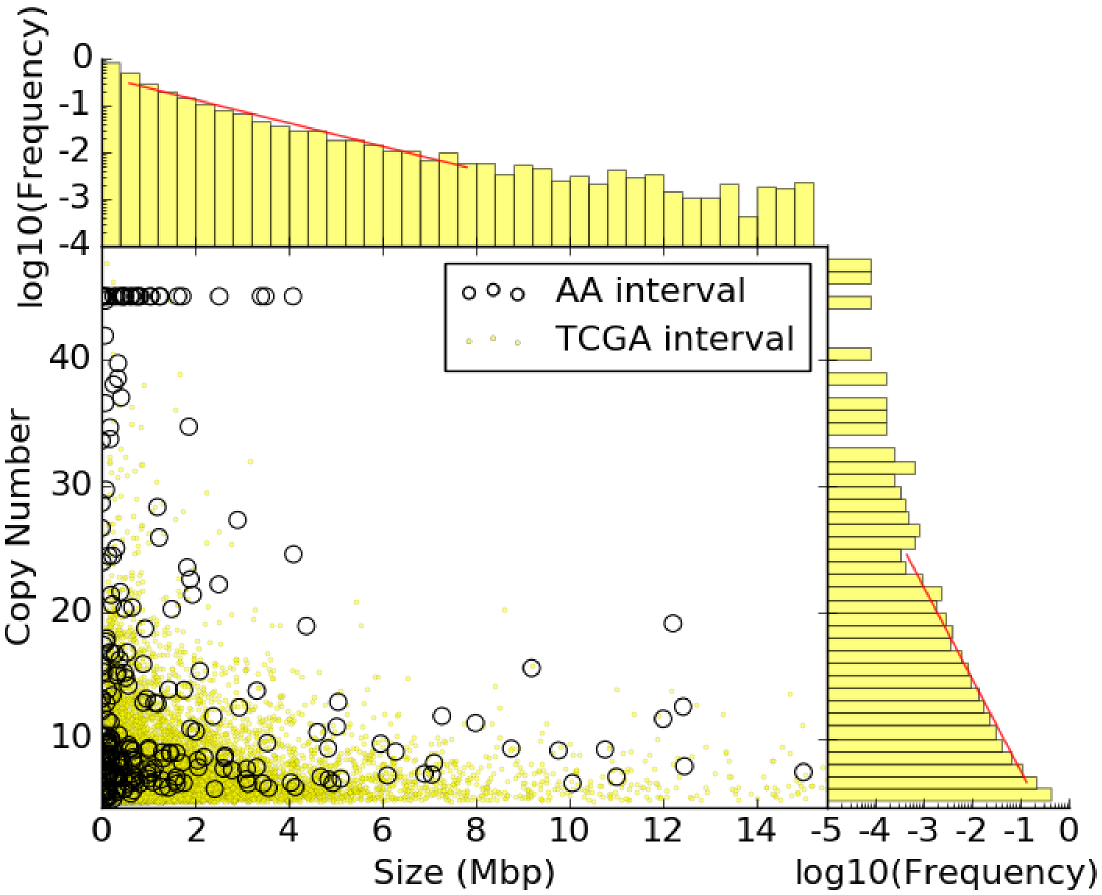

Figure S10: Distribution of amplicon intervals corresponding to Fig 2e: Scatter plot showing size and copy number distribution of individual intervals from all amplicons in sample set 1 (large empty circles) compared to distribution of intervals amplified in TCGA CNV array samples (yellow circles).

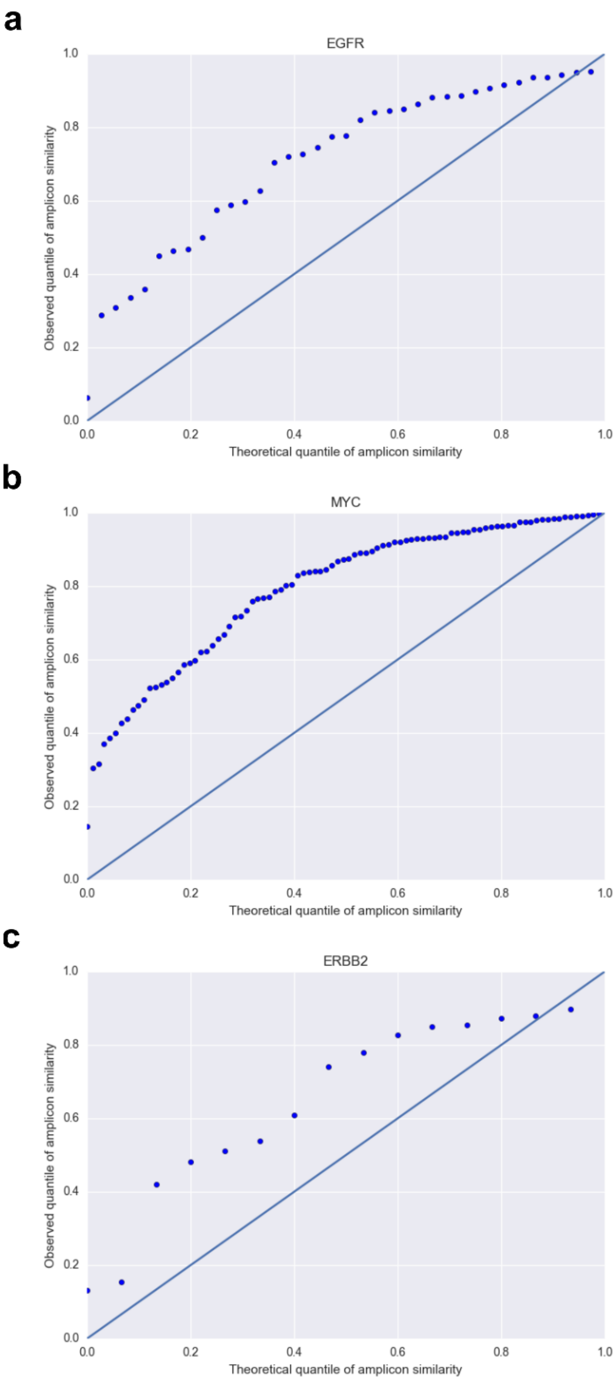

Figure S11: QQ-plot of similarity of overlapping amplicon intervals vs expected similarity: QQ-plots of pairwise-similarity (overlap as percentage of total length) of amplicon intervals containing 3 most frequently amplified oncogenes (a) *EGFR*, (b) *MYC* and (c) *ERBB2* as compared to expected percent overlap by randomly locating the intervals constrained on inclusion of the oncogene show that the amplicons intervals are randomly distributed around the oncogene and do not show enrichment of additional functional elements in the vicinity of the oncogenes.

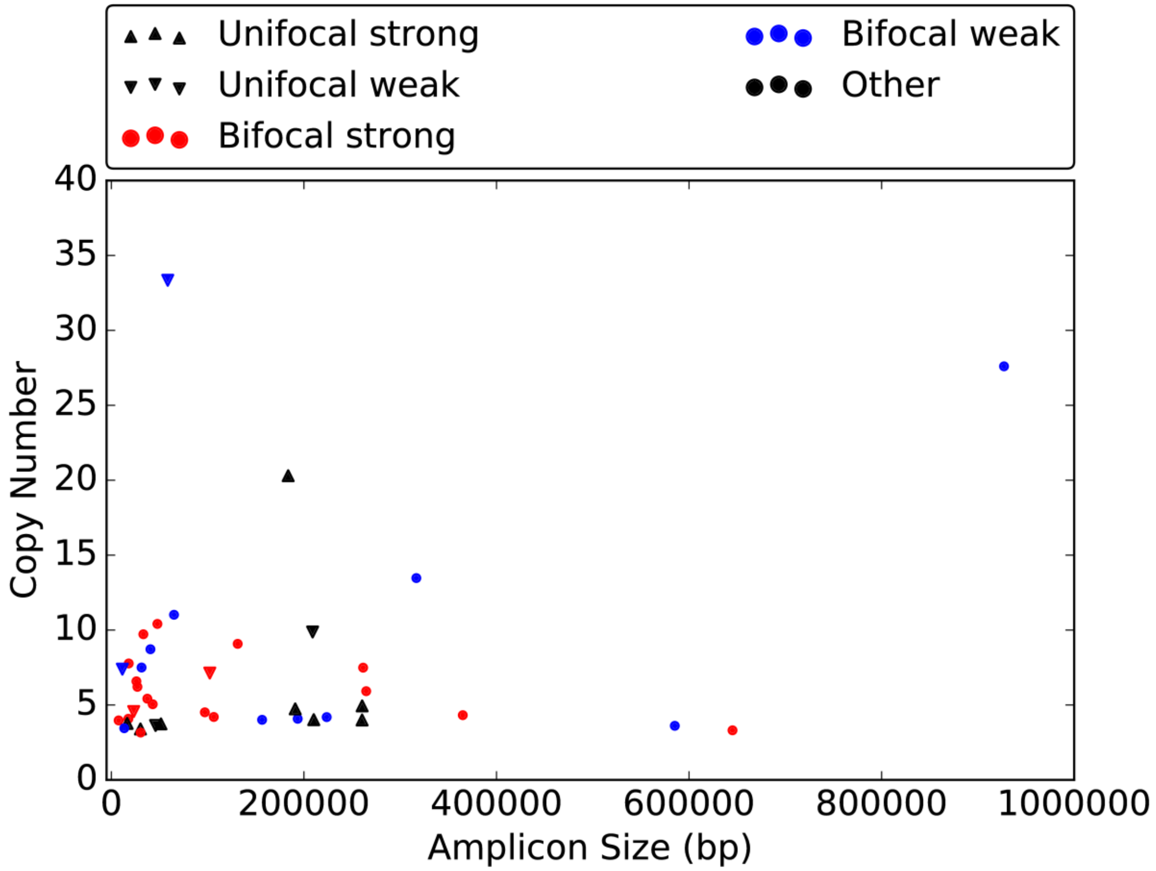

Figure S12: Size and copy number distribution of human-viral fusion amplicons in cervical cancer: Size and copy number distributions of fusion amplicons show that frequency of amplicons decreases both with size and copy number. Fusion amplicons are categorized as 'strong unifocal' signature (upward pointing triangles, 8 amplicons), 'weak unifocal' signature (downward pointing triangles, 6 amplicons), 'strong bifocal' signature (red markers, 19 amplicons) and 'weak bifocal' signature (blue markers, 12 amplicons). No clear separation is observed between the different categories.

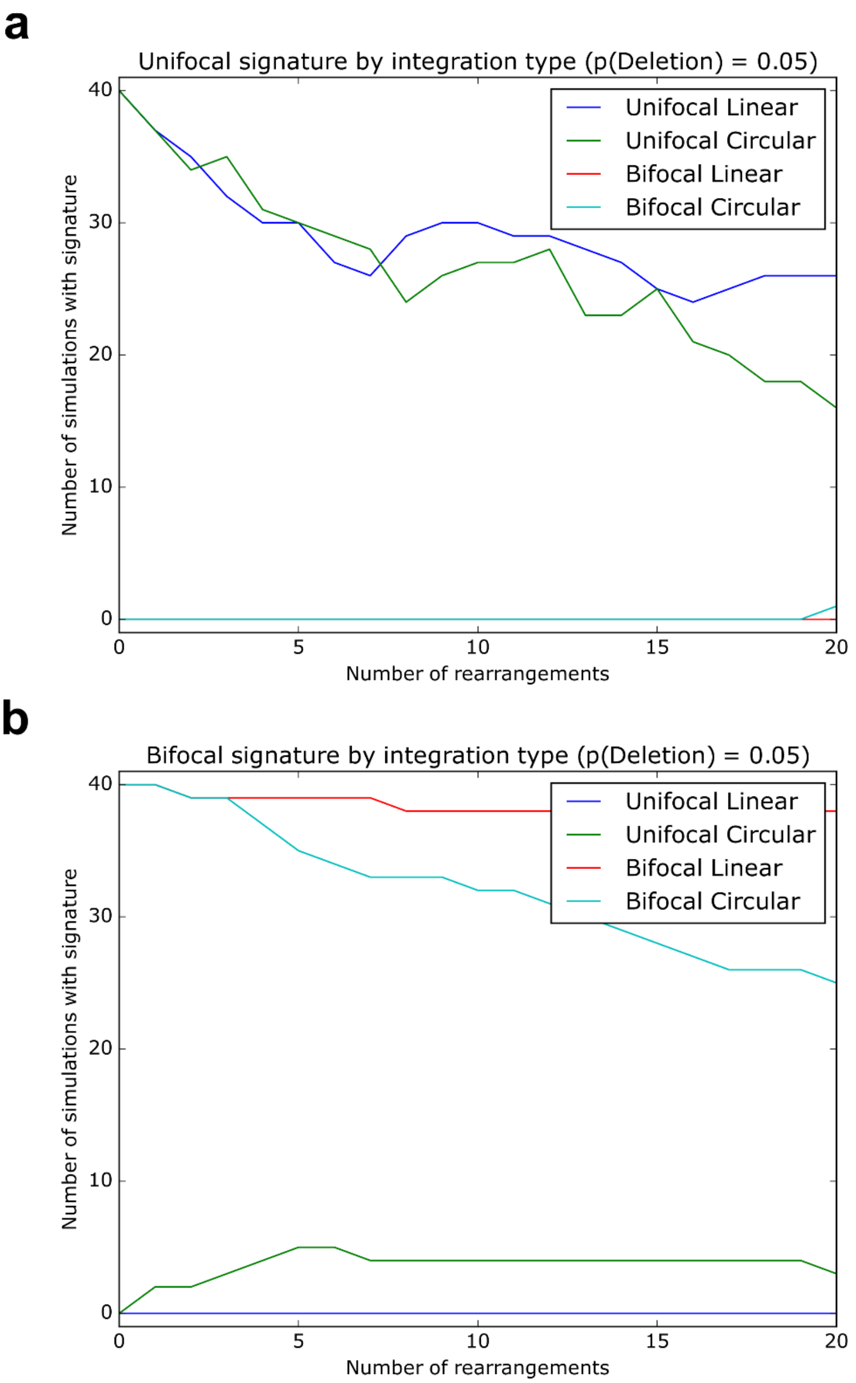

Figure S13: Unifocal and bifocal signatures over evolution of fusion amplicons: Evolution over 20 rearrangements of 160 simulated fusion amplicons, 40 each from 4 categories based on initial structure of viral insertion: (i) blue: intra-chromosomal unifocal insertion (e.g. A[BVC]D), (ii) green: unifocal insertion with circular extrachromosomal DNA formation (e.g. (BVC)), (iii) red: intra-chromosomal bifocal insertion (e.g. AB[VB]C) and (iv) cyan: bifocal insertion with circular extrachromosomal DNA formation (e.g. (BV)), shows that (a) amplicon structures originating from bifocal insertion do not show unifocal sequence signature or (b) amplicon structures origination from a bifocal insertion do not result show a unifocal signature.

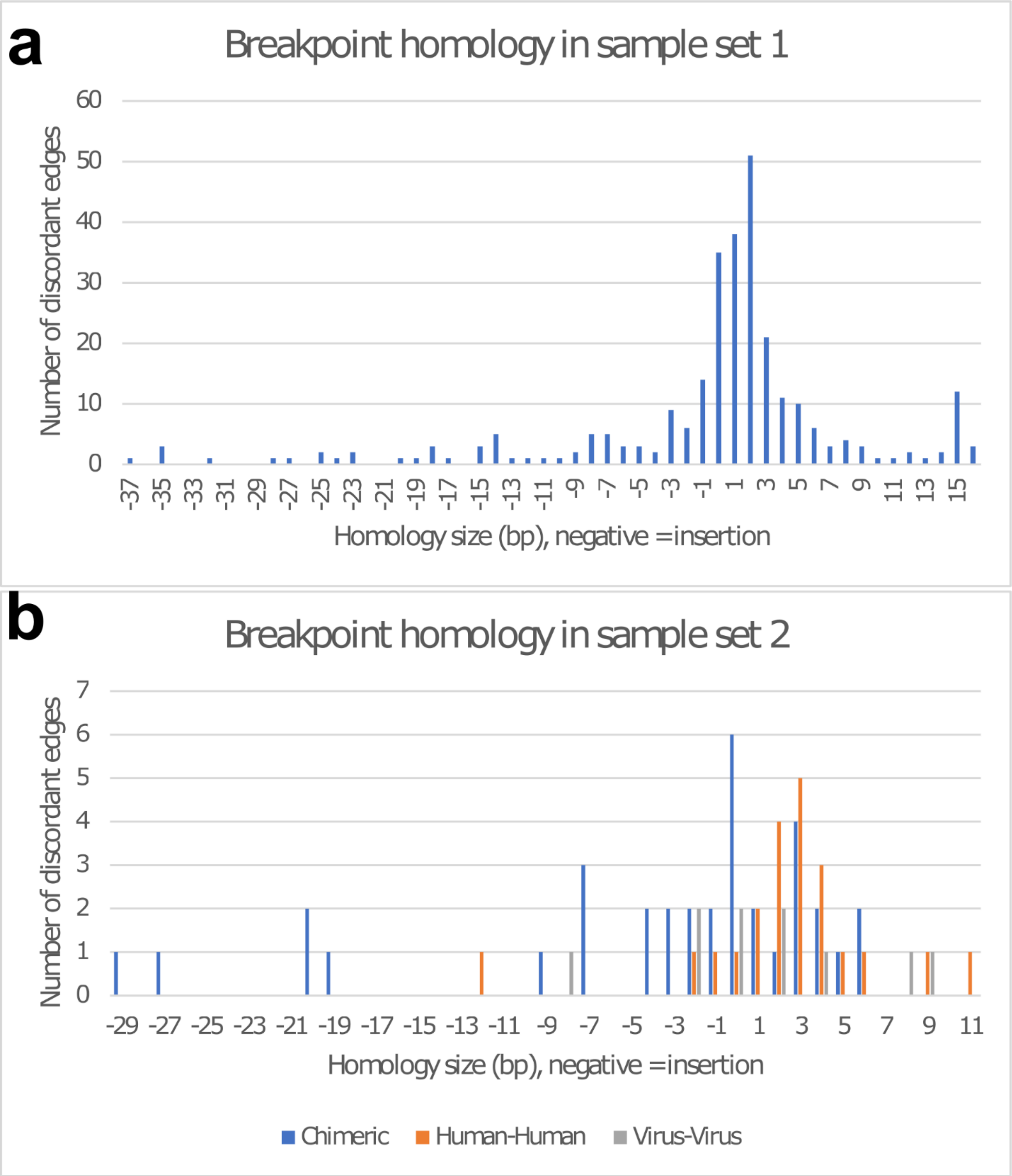

Figure S14. Breakpoint homology in amplicon rearrangements: Plots show the histograms of the distribution of length of insertions (negative) and homologous sequence (positive) at the discordant breakpoint edges. (a) Homology length/insertion size in edges in sample set 1 (pan-cancer), (b) Homology length/insert size in edges in sample set 2 (cervical cancer)

Section 1: Performance of AA on previously reported amplicons

We ran AA on previously reported amplicons and provide a comparison of AA reconstructions with previous studies. Here we present a comparison of the predicted amplicons by AA and the previous methods and contrast with the validated breakpoints.

ID	Source	Number of samples	Breakpoints reported by source	Structures reported by source
**1**	L'Abbate[1]	6 samples: Solid tumor cell lines with *MYC* amplificons	83 manually selected breakpoints, validated with Sanger sequencing. (134 predicted using Delly, Breakdancer, GASV, IGV, vectorette-PCR or long-range PCR)	Circos plots of structures predicted by manually walking through the breakpoints.
**2a**	Sanborn[2]	3 samples: GBM tissue	98 breakpoint edges predicted by BamBam out of which 42 were validated with PCR	Circos plots of structures by walking the breakpoint graph which used the 42 validated breakpoint edges.
**2b**	Dzamba[3]	3 samples: from **2a**	34 breakpoint edges inferred from the figures presented by the authors	CouGar visualization of the cycles using the 34 breakpoint edges
**3**	Akagi[5]	12 samples: HPV-infected cervical cancer tissue and cell lines	Human-viral chimeric edges arising from viral integration into human genome.	Visualization of putative structures, but not compared here because it was difficult to delineate the structures based on the visualizations presented in the manuscript.

<u>Dataset 1: 6 MYC amplicons:</u>

Dataset description: This dataset contained sequencing data for 6 samples (HL-60, GLC-1-DM, GLC-2, GLC-3, COLO320-DM, COLO320HSR) reported by L'Abbate et al[1]. Each sample was predicted by the original study to contain an amplicon with the oncogene *MYC*, along with PCR validation of breakpoint edges. We mapped the WGS samples to with with coverage between 4.6x to 10.5x and remapped the reads to hg19 reference genome with BWA MEM. We picked the seed intervals using the tool ReadDepth as described on Online methods section 1. We present a comparison between the list of breakpoints reported by the original study and AA as well as the predicted structures which used a portion of the breakpoints.

Breakpoint comparison:

We compared the breakpoints selected and validated by the authors of the original study and the breakpoints present in the top AA cycles ranked by copy number. The set of top cycles was selected as the minimal number of cycles which maximized the overlap with the breakpoint edges selected by the original authors. Out of 83 breakpoint edges validated by L'Abbate et al[1], 59 were

detected by AA. 15 edges were not reported due to intentional design of the AA algorithm: they either arose from small deletions comparable to the read insert size and hence ignored by AA or had very few supporting reads due to low copy number. 8 edges were only discovered by the original authors through PCR or IGV inspection. In many of these cases, AA reported the corresponding source edges thereby having minimal effect on the reconstruction. Finally, 1 edge was detected using Delly and was reported by AA as a source edge due to one end mapping outside the amplicon interval set. For this edge the original authors could not predict the span or structure of the new interval. This suggests that AA has sensitivity comparable to the manually curated set chosen from multiple SV calling tools. We cannot validate the 19 additional breakpoints detected by AA, however many of these breakpoints are accompanied by a change in coverage which gives us confidence that these predictions may actually be true.

Sample	Number of top AA cycles selected	AA BP edges	L'Abbate[1] BP edges	Common BP edges	Edges not reported by AA	Missed edges, SV size > 400bp & >10 reads	Missed edges, only found by PCR/IGV but not WGS
**COLO320-DM**	13	27	21	18	3	**1**	**0**
**COLO320-HSR**	10	18	18	12	6	**0**	**3**
**GLC1-DM**	5	11	16	8	8	**0**	**4**
**GLC2**	2	9	13	8	5	**0**	**1**
**GLC3**	1	4	5	4	1	**0**	**0**
**HL60**	2	9	10	9	1	**0**	**0**
**Total**	**32**	**78**	**83**	**59**	**24**	**1**	**8**

Comparison of predicted structures:

Sample, structure ID	Number of segments		AA cycles (cycle ranks)	AA = L'Abbate
	L'Abbate	AA		
**COLO320-DM, 1**	9+	27+	13 (1-13)	**No**
**COLO320-HSR, 1**	9+	18+	10 (1-10)	**No**
**GLC1-DM, 1**	7	9	1 (4)	**No (1 false edge and 1 missing edge)**
**GLC1-DM, 2**	15	14	4(1,2,3,4)	**No (1 false edge and 2 missing edges – same as above)**

**GLC1-DM, 3**	12	14	4(1,2,3,4)	**No (1 false edge and 2 missing edges)**
**GLC1-DM, 4**	1	1	1 (1)	**Yes**
**GLC2**	12	8	1(1)	**Yes***
**GLC3**	5	4	1 (1)	**Yes***
**HL60**	10	9	2(1,2)	**Yes***

* excluding small deletions < 400bp

Reconstruction description:

1. COLO320-DM:

In this sample, the authors proposed a single amplicon structure which contained 5 intervals, 2 from chr8 and 1 each from chr6, chr13 and chr16. The structure used 9 breakpoint edges but no evidence was presented for the connectivity between 2 edges. AA predicted 6/9 breakpoint edges and predicted the source edge for 2 more breakpoints. Notably, both studies predicted multiple other high copy rearrangements raising the possibility that the amplicon structure was more complex than one proposed by the original study.

2. COLO320-HSR:

For this sample, the authors predicted and validated the same set of breakpoints as COLO320-DM for both studies. In addition, AA predicted multiple new breakpoint edges which were not present in COLO320-DM suggesting that the cell line underwent additional rearrangements.

3. GLC1-DM, 1-3:

In this sample, the authors predicted 2 amplicons:

1. For amplicon 1, the authors proposed 3 structures (ID=1,2 and 3) containing 10 breakpoint edges. The top 4 cycles in AA contained 6 out of the 10 breakpoint edges and 1 false edge. All the 4 missed edges were also not detected by the authors using either Delly or Breakdancer and were only detected using IGV or PCR.

2. For amplicon 2, the authors proposed 1 structure (ID=4) which matched the prediction by AA.

AA predicted a 3rd circular amplicon on chr14 containing the oncogene *NKX2-1* with copy number 6.

4. GLC2:

In this sample, the authors proposed a single amplicon structure which contained 3 intervals from chr8 and used 9 breakpoint edges. Out of these 9 breakpoint edges, 8 were predicted by Delly or Breakdancer and 1 was predicted using IGV. AA predicted all 8 breakpoint edges and predicted source edges to match the 9th breakpoint predicted using IGV. The top 2 AA

cycles could be merged to matche the proposed structure where 1 breakpoint edge was replaced by the source vertex.

5. GLC3:

In this sample, the authors proposed a single amplicon structure which contained 3 intervals from chr8. The top AA cycle matched this structure.

6. HL60:

In this sample, the authors proposed a single amplicon structure which could be reconstructed by merging the top two cycles predicted by AA.

Dataset 2: 3 TCGA samples:

This dataset contained 3 glioblastoma samples (TCGA-06-0648, TCGA-06-0145, TCGA-06-0152) from Sanborn et al[2], also studied by Dzamba et al[3]. We down-sampled the bam files to coverage between 4×-7× by selecting read pairs with specific read group identifiers and aligned these reads to the hg19 reference genome using BWA MEM. The read group identifiers were selected to be sets of identifiers with the same read length and roughly similar insert length. The exact identifiers selected were as follows:

- TCGA-06-0145: 0, 0.1, 0.2 and 0.3; final coverage 5.2×
- TCGA-06-0152: 1, 2, and 3; final coverage 4.2×
- TCGA-06-0648: 0.9, 0.A, 0.X and 0.Z; final coverage 5.5×

We picked seed intervals based on calls from CNV calling tool ReadDepth with copy number > 5 and size > 100kbp as described in Methods 1A. Sanborn et al developed the tool BamBam to predict amplicon intervals and construct breakpoint graph. They presented structures predicted by walking the breakpoint graph. Dzamba et al developed the tool Cougar which algorithmically constructed the breakpoint graph and decomposed it into candidate structures. For interpreting the reconstructions of Cougar and BamBam, we inferred the amplicons as the connected components from the set of amplified structures where 2 structures are connected to each other if they share an overlapping interval.

Breakpoint comparison: AA vs Sanborn

We compared the breakpoint edges from the amplicon intervals common to both BamBam and AA and selected the minimal set of AA cycles in the same fashion as Dataset 1.

Sample	Number of top AA cycles selected	AA BP edges	Sanborn BP edges	Common BP edges	Edges not reported by AA	Missed edges, reads > 20% of highest read count in any edge
**TCGA-06-0145**	3	6	10	6	4	**0**
**TCGA-06-0152**	6	27	41	25	16	**0**

**TCGA-06-0648**	1	16	47	17*	30	**1**
**Total**	**11**	**50**	**98**	**48**	**50**	**1**

* One edge was, in fact, a combination of 2 edges separated by very short segments.

Breakpoint comparison: AA vs CouGar

Sample, Amplicon ID	Number of top AA cycles selected	AA BP edges	Number of top Cougar cycles selected	Dzamba et al[2] BP edges	Common BP edges
**TCGA-06-0145, 1**	1	3	1	1	1
**TCGA-06-0145, 2**	2	4	1	1	0
**TCGA-06-0152, 1**	1 (6th cycle)	3	1	2	1
**TCGA-06-0152, 2**	4 (7th-10th cycle)	24	2	18	18
**TCGA-06-0648, 1**	1	16	1	12	12
**TCGA-06-0648, 2**	2	0	1	0	0
**Total**	**7**	**50**	**6**	**34**	**32**

Comparison of predicted structures:

Sample, structure ID	Number of segments			AA cycles (cycle ranks)	AA = Sanborn	Sanborn = CouGar	AA = CouGar
	Sanborn	CouGar	AA				
**TCGA-06-0145, 1**	1	1	3	1 (1)	**Yes***	**Yes**	**Yes***
**TCGA-06-0145, 2**	-	1	3	2 (1,2)	**-**	**-**	**Yes***
**TCGA-06-0152, 1**	3	2	4	1 (6)	**Yes***	**No**	**No**
**TCGA-06-0152, 2**	22	18	24	4 (7,8,9,10)	**Yes***	**No**	**No**
**TCGA-06-0648, 1**	16	12	17*	1(1)	**Yes***	**No**	**No**
**TCGA-06-0648, 2**	-	1	2	2 (1,2)	**-**	**-**	**No**

* excluding small deletions < 10kbp and small segments < read insert size

Reconstruction description:

7. TCGA-06-0145:

 1. BamBam predicted a single amplicon containing a single interval and proposed 1 structure consisting of a single circular segment (+ 2 low copy structures). The top AA cycle matched the top cycle predicted by the authors and included with 2 additional small deletions. The structure predicted by CouGar was the same as BamBam.

 2. AA and CouGar predicted a 2nd amplicon containing CDK4 with copy number 13 which was not predicted by BamBam. AA predicted 3 segments out of which 2 segments were very small (104bp and 135bp). CouGar did not detect the 2 segments and instead predicted a direct connection between the end points of the 3rd large segment.

8. TCGA-06-0152:

 BamBam predicted 2 amplicons, and the authors proposed a single structure for each amplicon. CouGar reported 2 corresponding structures. The AA amplicon was unable to filter a repetitive interval which probably was not part of the amplicon. As a result, the first 5 cycles from AA consisted only of small segments (<10kbp) from the repetitive interval. We looked at cycles 6 through 10 which had much larger segments and copy numbers between 72-156.

 1. The first Sanborn structure contained 3 segments. The 6th AA cycle (CN=156) matched this but also included a small deletion on one of the segments. CouGar predicted a structure which included only 2 out of the 3 segments. CouGar missed the 3rd segment which was an inverted duplicate of the 2nd segment.

 2. The second Sanborn structure contained 22 segments. AA cycles 7 through 10 with CN between 72-97 cycles could be merged to obtain the proposed structured with 22 segments and 2 additional short deletions. However, these cycles could also be merged in alternate ways to obtain alternate structures. CouGar predicted 2 structures containing a total of 18 segments.

9. TCGA-06-0648:

 1. BamBam predicted 1 amplicon with 2 intervals and the authors proposed a single structure consisting of 16 segments. The top cycle in AA matched the proposed structure and included a small interval of size < 200bp. CouGar predicted a structure with only 12 segments.

 2. AA and CouGar predicted an amplified interval with CN~10 which was not reported by Sanborn. CouGar predicted a single linear segment with no breakpoint edges. AA predicted 2 linear structures separated by a region of low coverage but these structures did not contain any breakpoint edges.

<u>Dataset 3: 12 HPV-infected cancer samples:</u>

Akagi et al[4] studied sequence data of 12 HPV infected cancer samples (HNSCC and CESC) out of which they predicted 9 samples to contain HPV integration into the human (8 with HPV Type16 and 1 with HPV Type18). These predictions matched predictions by AA. Here we only compare against the chimeric connections reported by Akagi et al since they predicted the structures on a per sample level through chromosomal walking and exact breakpoints and order of segments were difficult to infer based on the visualizations in the manuscript. The full reconstructions are available on figshare. The viral integration sites predicted by AA exactly matched those predicted

by Akagi et al in 6 out of 9 samples. In sample UM-SCC-47, AA predicted 7 chimeric breakpoint edges with 1 end connected to human genome and the other to the viral genome whereas Akagi et al predicted 6. In samples UPCI:SCC090 and CaSki, AA predicted 28 and 44 chimeric edges respectively as compared to 33 and 48 by Akagi et al, however all 9 edges missed by AA had 5 or less reads suggesting that these edges had a low copy number.

1. L'Abbate, A. *et al.* Genomic organization and evolution of double minutes/homogeneously staining regions with MYC amplification in human cancer. *Nucleic Acids Res.* **42,** 9131–9145 (2014).
2. Sanborn, J. Z. *et al.* Double minute chromosomes in glioblastoma multiforme are revealed by precise reconstruction of oncogenic amplicons. *Cancer Res.* **73,** 6036–6045 (2013).
3. Dzamba, M. *et al.* Identification of complex genomic rearrangements in cancers using CouGaR. *Genome Res.* **27,** 107–117 (2017).
4. Akagi, K. *et al.* Genome-wide analysis of HPV integration in human cancers reveals recurrent, focal genomic instability. *Genome Res.* **24,** 185–199 (2014).
