# Supplementary material for: Exploring the landscape of focal amplifications in cancer using AmpliconArchitect
Source: Nat Commun. 2019 Jan 23;10:392. doi: 10.1038/s41467-018-08200-y (PMC6344493; doi:10.1038/s41467-018-08200-y)
Supplement: Supplementary file 2 — Description of Additional Supplementary Files [file 41467_2018_8200_MOESM2_ESM.pdf]

Description of Additional Supplementary Files

Supplementary Data 1 :

Sample list for sample set 1

Supplementary Data 2 :

Comparison of germline and somatic amplified intervals in TCGA

Supplementary Data 3 :

Seed intervals selected in sample set 1

Supplementary Data 4 :

Final amplicon intervals, oncogenes and classification (single or multi-interval)

Supplementary Data 5 :

AA cycles vs FISH comparison

Supplementary Data 6 :

Oncogene amplified in corresponding types in TCGA and sample set 1

Supplementary Data 7:

Viral sample list with detected HPV strain and integrations

Supplementary Data 8 :

List of viral amplicons, oncogenes and classification (unifocal/bifocal)
